# Alternative Protein-Protein Interfaces Are Frequent Exceptions

**DOI:** 10.1371/journal.pcbi.1002623

**Published:** 2012-08-02

**Authors:** Tobias Hamp, Burkhard Rost

**Affiliations:** 1 TUM, Bioinformatik - I12, Informatik, Garching, Germany; 2 Institute of Advanced Study (IAS), TUM, Garching, Germany; 3 Department of Biochemistry and Molecular Biophysics, Columbia University, New York, New York, United States of America; National Cancer Institute, United States of America and Tel Aviv University, Israel

## Abstract

The intricate molecular details of protein-protein interactions (PPIs) are crucial for function. Therefore, measuring the same interacting protein pair again, we expect the same result. This work measured the similarity in the molecular details of interaction for the same and for homologous protein pairs between different experiments. All scores analyzed suggested that different experiments often find exceptions in the interfaces of similar PPIs: up to 22% of all comparisons revealed some differences even for sequence-identical pairs of proteins. The corresponding number for pairs of close homologs reached 68%. Conversely, the interfaces differed entirely for 12–29% of all comparisons. All these estimates were calculated after redundancy reduction. The magnitude of interface differences ranged from subtle to the extreme, as illustrated by a few examples. An extreme case was a change of the interacting domains between two observations of the same biological interaction. One reason for different interfaces was the number of copies of an interaction in the same complex: the probability of observing alternative binding modes increases with the number of copies. Even after removing the special cases with alternative hetero-interfaces to the same homomer, a substantial variability remained. Our results strongly support the surprising notion that there are many alternative solutions to make the intricate molecular details of PPIs crucial for function.

## Author Summary

The number of known protein-protein interactions (PPIs) grows rapidly, yet their molecular details remain largely unknown. Over the last years, structural biologists have addressed this issue with an increased output of structurally resolved hetero complexes. This wealth now enables statistically significant quantitative statements about interface properties. Here, we addressed the question how interfaces differ when observing the same proteinprotein interaction twice. A new dataset derived from the entire PDB was analyzed employing different definitions for the “same interaction” and a range of interface similarity measures. The hypothesis was that the interface between the same pair of proteins stays the same irrespectively of how often it is measured. Although the results mostly confirm this hypothesis, the surprising finding was how often it was not true: for many comparisons of interfaces, the molecular details of the interaction differed importantly, often without the slightest change of amino acids. In addition, no matter how much “special cases” were sieved out, the essential message remained: interfaces appear immensely plastic. Hand-selected sample structures largely support this view. In general, we complement a series of recent studies focusing either on family-family interactions or exploring other aspects of protein-protein complexes.

## Introduction

### PPIs in high-resolution reveal molecular details of network edges

The study of high-resolution three-dimensional (3D) structures of proteins as deposited in the PDB, the Protein Data Bank [1], began with peptides [2], [3] and has increasingly included larger complexes of interacting proteins [4]. These complexes, or PPIs (Protein-Protein Interactions), capture the molecular details of interaction networks. The network view, in turn, has become increasingly important for, e.g., the ranking of genes according to their probability of being causative for a particular disease [5]–[7] as needed for Genome-wide Association Studies (GWAS).

Despite this wealth of high-resolution interaction data, the set of interactions for which the exact molecular mechanisms are known remains immensely incomplete [8] and with it experimental and computational descriptions of binding positions and binding-induced conformational changes [9]–[11]. Nevertheless, studies of available structures have shown that related proteins have similar binding sites [12], that permanent and transient interactions differ so substantially from each other [13] that PPI hotspots can be predicted from sequence [14], [15], and that we can accurately distinguish between specific and unspecific contacts [16]. Many others have addressed related tasks [16]–[23], including even the contribution of water to the binding modes of PPIs [24].

### We study external PPIs from many new perspectives

An excellent recent work reviews various types of protein interactions [25]. We want to complement it with a quantitative analysis of the interface variability of external interactions, i.e. interactions between two protein chains coming from different genes. These typically correspond to the edges in a PPI network. The atomic structures of their interfaces often seem to cluster into particular architectures [16], [26] and it has been suggested that they are conserved within and between organisms [26]–[31]. Many authors have also analyzed the molecular details of binding within and between their domain families [32]–[37].

For example, they found that two different SCOP domain families exhibit more than one orientation of binding about 24% of the time [33]. Beside this number, however, only few more details were given about the underlying biological variety and in particular the causes of differential interfaces. The problem we see with this approach is that members of a SCOP family only share similar 3D structures and that the observed variability in binding might simply be explained by sequence variation. In fact, the inference of similarity in structure (homology modeling) is much more accurate than the inference of protein-protein interactions [26]. So far no studies based on significantly sized data sets have addressed the question to which extent the interface between two different proteins is biologically conserved, i.e. excluding diversity due to sequential differences.

Another challenge for the analysis of large-scale data sets have been crystal contacts and the difficulties of automated methods to correct such problems (e.g. the PQS [38] or the PISA [39] service). Authors “addressed” these problems by either entirely excluding different interfaces suspecting that those originated from non-biological contacts, or by leaving it open to which extent their results might have been created by such contacts.

Here, we address both issues. First, we realized that the number and quality of author-assigned biological assemblies in the PDB now suffices to enable a quantitative study like this one. For the large majority of entries, the PDB now provides biologically relevant structures from the crystallographers themselves. Similar to PQS or PISA, they describe a complex as it occurs in the living cell. At the same time, however, they are more accurate and easier to verify than *de novo* predictions. Therefore, we did not discard any high-resolution complex or interface therein.

**Figure 1:**
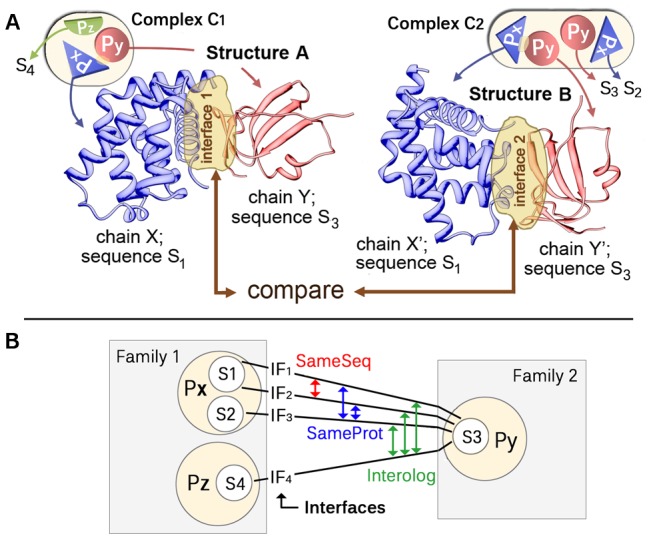
(**A**) **Sketch for interface comparison.** Two proteins Px and Py always interact in the same way, do they? We compared pairs of proteins for which we found several experimental solutions for their interaction. Assume that we have two high-resolution protein complexes C1 and C2. From these, we pick two hetero-dimers (Structure A and Structure B) for the interaction between proteins Px and Py (identified by the chains X and Y in Structure A, and by X′ and Y′ in Structure B). We then compared the interface of the same interaction between those two experimental solutions. (**B**) **The PPI network induced by complexes C1 and C2.** Complexes C1 and C2 contain two protein-protein interactions: Px-Py and Px-Pz. We differentiated between three types of interface comparisons. First, we only compared interactions corresponding to same pair of sequences (SameSeq; red; shown in A). Then, sequences could change as long as the original proteins remained the same (SameProt; blue; interfaces S1/S3 are compared to S2/S3; both sequences S1 and S3 are variants of protein Px). Finally, we compared interologous interactions (green; interfaces Px/Py are compared to Pz/Py; Px and Pz come from the same family).

Secondly, put most extremely, we ask the question: if X-ray crystallographers measure the same interaction twice, do they get the same result? The main focus is first on the variability of the interaction between identical variants of the same two proteins (*SameSeq*). In other words, we look at external interactions corresponding to the same pair of protein sequences and estimate how often the interfaces are different (Fig. 1A; Fig. 1B: the red arrow compares two sequence-identical interactions). We then extend our analysis by allowing minor sequence variations in corresponding interactors (e.g. in the form of point mutations; *SameProt*). However, we still maintain the comparison between essentially the same proteins, because we make sure that a sequence change does not go hand-in-hand with a change of the original protein (Fig. 1B: for the blue interface comparisons, the sequences have changed [S1/S3 vs. S2/S3], but the original proteins [Px/Py] remained the same). Finally, we compared two external interactions corresponding to the same family pair, i.e. “interologs” (*Interolog*). In a dimer-dimer comparison on this Interolog-level, corresponding interactors still had a similar 3D structure, but their sequences could be very different. (Several authors have been using the term “interolog” [40], [41]; it has the advantage over the term “homolog” that no evolutionary relation is implied in the definition; Fig. 1B, green: interfaces between proteins Px and Py are compared to those between Pz and Py).

## Methods

### PPI (protein-protein interaction) data set from the PDB

Each node in a PPI network typically refers to a UniProt [42] entry. While UniProt stores information about proteins, its first layer of organization is genetic: every entry corresponds to a unique location on a genome. Hence, in order to find reliable structural evidence of PPI network edges, we mined the PDB [43], [44] for interacting proteins which map to different Uniprot/Swiss-Prot [45] identifiers. We extracted such external protein-protein interactions (i.e. interactions originating from two different genes) in the following way: first, we downloaded all author assigned biological assemblies from the PDB. We then retained only X-ray structures that had a resolution <2.5 Å and mapped to at least two different UniProt/Swiss-Prot entries (author assignment available for 99% of all such structures). We primarily used the PDB< = >Swiss-Prot mapping provided by the PDB and only performed the following step if this mapping was not available: we BLASTed [46] the PDB SEQRES sequence (at least 30 residues long) against the Swiss-Prot database, thresholding at E-Values <10-3 and requiring at least 90% of the PDB chain to be aligned. (When we found more than one hit, we took the one with the lowest E-Value; when we had none, we discarded this complex.) Having found those ‘interesting’ complexes, we extracted all interacting pairs of chains pointing to two different Swiss-Prot entries. At this early stage of our procedure, we only required one pair of atoms of the two chains to be closer than 0.6 nm (6 Å) in order to consider them interacting.

Note that in an earlier version of this work, we had exclusively used the PISA service [39] to obtain biologically relevant assemblies. In Section S3.3 in Text S1, we give reasons why we switched to author assigned complexes, an accuracy estimation of PISA in the context of hetero-complexes and other results compiled with the PISA based data set.

### Definition of PPI interfaces

Having found all structures of external interactions, we annotated their interfaces. Given a hetero-dimer with chains X and Y (X and Y come from different genes), we considered a pair of residues Rx and Ry as part of the interface if it contained at least one pair of atoms closer than 0.6 nm (6 Å) or if it met all three conditions: (i) both residues changed their accessible surface area upon binding (dASA: replacing the binding partner by water), (ii) Rx had no other interaction partner within 0.6 nm (6 Å), (iii) of all residues in protein chain Y that changed their accessible surface area (ASA), Ry was the closest to Rx. The latter included interactions that fell slightly above the 0.6 nm (6 Å) threshold but should still be considered interacting by their ASA change (we present a brief analysis of the effect of including dASA in the interface annotations in Section S3.1 in Text S1). We annotated each interface residue by two structural descriptors: dASA and d reflecting the distance (in Ångstrøm of the closest binding residue). Having defined all interface residues, we removed each hetero-dimer with fewer than five interacting residues on either chain from our data set. Finally, we assigned each remaining hetero-dimer its “interface copy number”. To this end, we first determined the original complex a hetero-dimer was extracted from. Then we counted how many other hetero-dimers were also extracted from this complex and had exactly the same two SEQRES sequences as the hetero-dimer under consideration. This “interface copy number” was assigned to all these sequence-identical hetero-dimers of the complex (Section S5 in for details).

### Measures for face and interface similarity

Overall, we applied nine different interface similarity measures to our data, covering various types of changes. They are defined in detail in Section S2 in Text S1. The variety of these measures guaranteed that we captured as many aspects of “interaction similarity” as possible. We found significant differences between these measures, but with respect to our overall conclusions, we considered it more important to eschew obfuscation than to present all necessary details. Therefore, we used only the two most representative and intuitive measures in the main text, namely the Face Position Similarity and the L_rms. In the following, we refer to “interface” as all the residues that “touch each other” between two interacting proteins (Fig. 1), and as “face” as all the residues on one side of the interface. Also note that we always reduced hetero-dimers to common residues before comparing their interfaces. Please see Section S2 in Text S1 for details of this procedure.

#### Face Position Similarity

The Face Similarity tries to measure the conservation of *face* residues in both interfaces. For instance, assume that residues 1,2,3 interact on X, and residues 1,3,7,8 on X′ (Fig. 1). The size of the intersection is then 2 (1, 3), i.e. the two faces on X and X′ have two residue positions in common (residues 1 and 3). The average face size is 3.5 = sqrt(3*4) (geometric mean) and the Face Position Similarity for X-X′ becomes 2/3.5. The calculations of the same number for the pair Y-Y′ yielded two values of Face Position Similarity. Among all the measures that we tried, the Face Position Similarity represented a good average interface similarity. Other measures were either more robust against smaller changes (e.g. Sphere Radius Ratio) or more sensitive, e.g., in terms of rotations (Interface Position Similarity) or side chain movements (Convex Hull Overlap).

#### L_rms

Most notably used in the CAPRI [47] experiments, this measure first optimally superimposes the two larger proteins under consideration (‘receptors’) and then applies this transformation to the two smaller proteins (‘ligands’). The classical Root Mean Square Deviation (RMSD) of the backbone atoms of the ligands is the L_rms. Note that this approach differs substantially from the other measures tested. Firstly, it returns a distance in Å instead of a similarity between 0 and 1. Secondly, it measures the displacement of the entire protein, not only the interface. Conformational changes of the ligands can lead to high distances as well as different binding positions. We still used this measure in the main text in order to link our results to related work. Cross-correlations to other measures are defined and presented in Sections S1.4 and S6.3 in Text S1.

### Grouping interfaces

Before we could apply the interface similarity measures to our entire collection of external interactions, we needed to group the structures so that we could differentiate between (and not mix) different types of sequence divergence. This also addressed the redundancy immanent in the PDB in the form of, e.g., overrepresented protein families. We hierarchically clustered the hetero-dimers over three levels, corresponding to increasing levels of sequence divergence (a more technical description of the following procedure can be found in Section S1.1 in Text S1)

First, we assigned two hetero-dimers to the same Level SameSeq group if they corresponded to same pair of SEQRES sequences (Fig. 1B: we add interfaces 1 and 2 to the same Level SameSeq group; other interfaces become single member Level SameSeq groups). Next, we reduced the influence of over-represented proteins. This was achieved by adding Level SameSeq groups to the same Level SameProt group if they corresponded to the same pair of associated Swiss-Prot identifiers (Fig. 1B: Level SameSeq groups S1-S3 and S2-S3 go into one Level SameProt group, S3-S4 to another). Clusters obtained in this way should represent the classical notion of edges and nodes in a PPI network. Our final Level Interolog addressed overrepresented families: we merged Level SameProt groups that pointed to the same pair of Pfam [48] families into one Level Interolog group (Fig. 1B: both Level SameProt groups are merged into one Level Interolog cluster; Fig. S1 in Text S1 for a graphical illustration of the clustering).

### Interface similarity distributions

Without the grouping above, any distribution of pairwise interface similarities would have been highly dominated by large and well-studied complexes for which many structures are available. Avoiding this bias demanded to group PPIs differently (Levels SameSeq to Interolog) and also to embrace this alternative grouping when trying to infer biologically meaningful similarity distributions. While the following procedure successfully reduced the bias stemming from overrepresented sequences and sequence families, we deliberately left Level SameSeq clusters unchanged in the assumption that all binding modes are biologically meaningful and that eliminating this redundancy would remove more biology than noise (please see Section S1.2 in Text S1 for a more mathematical description of the following procedures).

#### Distribution D-SameSeq

The interface similarity distribution in a SameSeq group describes the variety of the binding modes of sequence-identical pairs of proteins (Fig. 1A; Fig. 1B: red). We calculated this similarity distribution by using all pairwise interface similarities of the members of a SameSeq group: we estimated the probability that a similarity falls into a particular similarity range (e.g. 0.0 to 0.1) and repeated this for all similarity ranges. SameProt groups contain several SameSeq groups. Thus, for a SameProt group distribution, we averaged over the distributions of all its SameSeq groups. Correspondingly, Interolog distributions originate from averaging over the distributions of the member SameProt groups. Essentially, the above leaves us with many Interolog distributions. For a view of all those distributions, we simply averaged over all Interolog distributions to obtain distribution D-SameSeq. It can be interpreted in the following way: First, we randomly choose a family pair (Interolog group; e.g. globin - globin). From this family pair, we randomly pick a Swiss-Prot pair (SameProt group; e.g. bovine hemoglobin-A – bovine hemoglobin-B) and then a sequence pair (SameSeq group; e.g. wild-type bovine hemoglobin-A – wild-type bovine hemoglobin-B). Distribution D-SameSeq then gives the chance that the similarity between two interfaces of this sequence pair (‘two observations’) lies in a specific similarity range. For example, D-SameSeq may define the probability that two interfaces have a similarity between 0.1 and 0.2 to be 7%. Note that the role of SameProt and Interolog groups here is simply to reduce sequence redundancy. We are still only comparing interfaces between sequentially identical protein pairs. By leaving out Level Interolog for example, we would highly bias D-SameSeq towards the globin family. The PDB not only contains many structures of one particular hemoglobin variant, but of many variants (for example from different organisms). Only by combining all these variants in one Interolog group and giving the distribution of this group the same weight as any other group, we limit the influence of hemoglobin. Similarly, combining SameSeq clusters in one SameProt cluster makes sure that one hemoglobin variant does not suppress the influence of others.

#### Distribution D-SameProt

Distribution D-SameSeq compared observations of sequentially completely identical PPIs (Fig. 1A; 1B: red). This provided information about native interface variability, i.e. variability coming from environmental and conformational changes or simply different energetic minima between two proteins. The edge in a PPI network, however, allowed for some sequential variation as a node only referred to a gene, not to a specific gene product (Fig. 1B). The distribution D-SameProt revealed how such modifications affected interface variability. First choose a family pair and then a Swiss-Prot pair. This yields several sequence pairs (SameSeq clusters) that all map to the same edge in a PPI network (Fig. 1B: S1/S3, S2/S3). Pick two of these pairs and compare all of their interface structures to derive a similarity distribution (Fig. 1B: blue). For example, we compared all wild-type bovine hemoglobin interfaces to those where residue 90 of subunit A was changed from H to Y (natural variant of bovine hemoglobin-A; P01966). Repeating this for all sequence pairs (e.g. for all bovine hemoglobin variants) we calculated many distributions. Averaging them yielded the overall Level SameProt distribution (e.g., the average interface variability of bovine hemoglobin). From here on, the same procedure as for D-SameSeq applies: calculate all Level SameProt distributions of the parent Interolog cluster (e.g. distributions for all globin-globin clusters, not only bovine hemoglobine). Average over these to obtain the Interolog distribution; calculate and average all Interolog distributions. This yields the overall distribution D-SameProt. Put simply, it reflects the chance that two interfaces from the same PPI edge lie in a particular similarity range, given small sequence changes have occurred.

#### Distribution D-Interolog

Finally, we want to investigate the diversity of binding modes between proteins from the same family pair, but different gene pairs (Fig. 1B: Px/Py, Pz/Py; green). The procedure to derive this overall similarity distribution D-Interolog is analogous to that for D-SameProt: first, choose a family pair (e.g. cyclin - protein kinase) and two of its Swiss-Prot pairs (e.g. cyclin E1 – protein kinase 2 and cyclin B1 – protein kinase 2). Then, pick one sequence pair from each Swiss-Prot pair (e.g. the wild-type variants), compare all of their interfaces and calculate the Level SameSeq distribution. Repeat this for all sequence pairs from the two Swiss-Prot pairs and obtain the Level SameProt distribution by averaging over all Level SameSeq distributions. This again is repeated for all possible Swiss-Prot pair combinations in order to derive the Level Interolog distribution. Finally, average over all single Level Interolog distributions in order to derive the overall distribution *D-Interolog*. Put simply: randomly choosing a pair of interacting families and then two of its protein pairs, D-Interolog gives the chance that a typical comparison of their interfaces will result in a particular similarity. (Note that this procedure quickly leads to unfeasible amounts of interface comparisons. We have therefore limited the number of protein pairs per family and the number of sequence pairs per protein pairs to a maximum of 50.)

### Analyzing the influence of homo-oligomeric assemblies

**Figure 2:**
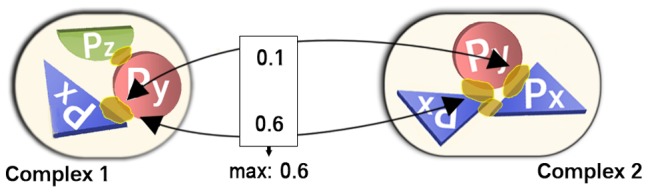
Filtering out interface diversity introduced by homomers. Assume you want to compare an interface Px-Py in complex 1 to the interfaces in complex 2. Usually, you will calculate two similarities (0.1 and 0.6), because there are two Px-Py interfaces in complex 2. Looking for homomers, you will find the two sequence identical Px chains in complex 2 interacting and connecting the two Px-Py interfaces. Now, you can correct the comparison by using only the one best match (0.6). The comparisons of the “worse” alternatives are discarded.

The same proteins may aggregate to form a homo-oligomer and bind as such a complex to another protein. In this case, the other protein often “sees” different parts of the homomeric chain, resulting in very different external interfaces. For example, a homo-dimer with chains X_1_ and X_2_ might bind to another chain Y with two different interfaces (Fig. 2). Hence, we will have two hetero-dimers X_1_/Y and X_2_/Y with low interface similarity. As it can be argued whether both of these interfaces should be considered as one big interface or treated separately (Discussion), we analyzed their influence on the distributions D-SameSeq to D-Interolog. To this end, we defined homo-oligomers in two different ways. Firstly, we used the classical notion, namely that all chains of a homomer have the same SEQRES sequence. Secondly, we introduced “structural homomers” as interacting chains from the same family. This corresponded to all complexes that *look* homo-oligomeric on a structural level (low RMSD; Fig. S4B in Text S1), but can differ in sequence.

Consequently, when comparing two interfaces X/Y and X′/Y′ from two different PDB entries, it was checked whether or not one of the chains X′ and Y′ were part of homo-oligomers (i.e. whether there were homomers X′/X′_1_/…/X′_n_ or Y′/Y′_1_/…/Y′_m_) and whether or not these homo-oligomers had other external interfaces with the same interaction partner as in X′/Y′ (i.e. whether X′ had interfaces with Y′_1_/…/Y′_m_ or Y′ interfaces with X′_1_/…/X′_n_). Having identified the set of all those sequence- or family-identical interfaces (including X′/Y′), they were compared to X/Y. Only if X/Y< = >X′/Y′ was the best match among all alternatives, the corresponding similarity was used. Otherwise, the entire comparison was discarded (Fig. 2.)

Eventually, the roles of X/Y and X′/Y′ are switched, and the procedure is repeated because all interfaces are compared with all others in the distributions D-SameSeq to D-Interolog. In this way asymmetries arising from only considering the oligomeric context of one side of the comparison were filtered out.

We applied “structural homomerization” only in the context of D-Interolog. For the two other distributions, it would have led to comparisons of interfaces between different protein pairs, thereby invalidating the constraints of these distributions. Also note that the above definition only allowed for comparisons of interactions between two different families.

## Results

### We calculated three non-redundant interface similarity distributions

Our complete data set of external protein-protein interactions (PPIs) comprised 37,338 hetero-dimers. We grouped and filtered them on three different levels with decreasing sequence redundancy (Methods). For instance, the first clustering level had 634 groups that contained sequence-identical hetero-dimers from at least two different high-resolution PDB entries. We compiled various statistics on this data set, including the distribution of cluster sizes on each clustering level, of oligomeric states, interface sizes and even of the conservation of protein function (Section S3.2 in Text S1).

In order to capture different facets of interface similarity, we introduced and evaluated nine different similarity measures (Sections S2 and S6 in Text S1). In the following, we focused on the results from two measures (Face Position Similarity and L_rms; Methods), and reported only qualitative findings for the others. The first measure (Face Position Similarity) was most representative for all other seven measures while the second (L_rms) enabled direct comparisons of our results to related work, e.g. to the CAPRI [47] experiments. For each measure, we used our clustering to calculate three different interface similarity distributions, corresponding to increasing levels of sequence divergence between interactions (D-SameSeq to D-Interolog [Methods]). These distributions constitute the main result of this work and are presented in the following. They were calculated such that all proteins and families of our data set contributed equally, regardless of their respective over-representation in the PDB. Finally, we measured how the distributions change when excluding the interface variability introduced by a protein binding differently to the same homomer. We give a short summary of this after the presentation of the unmodified distributions.

### Most interfaces were mostly robust for identical pairs of interactions (D-SameSeq)

**Figure 3:**
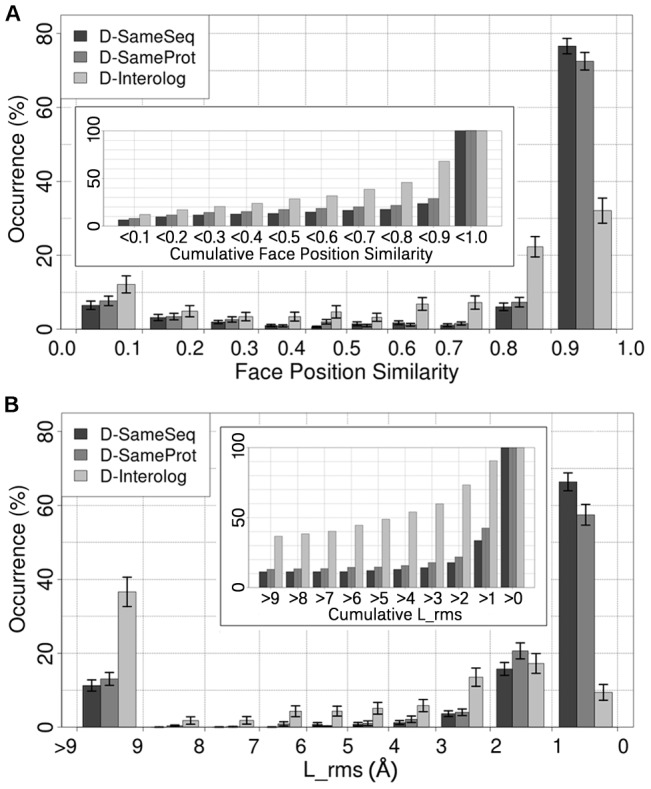
Faces are similar yet different. For two different ways to measure interface similarity - Face Position Similarity [**A**] and L_rms [**B**] - we present the similarity distribution for all interfaces. The rightmost interval shows largely identical faces, the leftmost completely different faces. For each similarity range and measure, there are three bars: one for each type of sequence divergence (D-SameSeq to D-Interolog). For example, Face Position Similarity finds about 7% of all the interface similarities at D-SameProt to fall in the range 0.0–0.1, i.e. suggests in 7% of the cases completely different outcomes when experimentally measuring the same interaction again. The error bars show standard errors and are explained in Section S1.3 in Text S1. The inlet displays the cumulative distribution giving the fraction of all similarities that differ by a certain value. For instance, 21% of all interface comparisons result in a value above 2 Å according to the L_rms in D-SameProt. In these cases, the two smaller proteins are clearly not in the same position after superimposing the two larger proteins.

When two different experiments measured exactly the same external interaction (distribution D-SameSeq; Methods), usually the interface between the two proteins was identical. Depending on the similarity measure, the number of largely conserved interfaces varied between 60 and 89% (Fig. S3 and Fig. S9 in Text S1). The most representative measure (Face Position Similarity) found the same interface in 75–79% of all cases (Fig. 3A, D-SameSeq, rightmost bar). Conversely, interfaces largely differed between two observations in about 12% (Fig. 3A, Face Position Similarity <0.5).

Other measures introduced in this work were, for instance, very sensitive to side-chain movements (Convex Hull Overlap), or only roughly assessed the conservation of the interface location (Sphere Radius Ratio). Taking into account two similarity measures simultaneously, small differences were observed in as many as 49% of all comparisons (Section S6.3 in Text S1).

In contrast to our measures, the L_rms (used by CAPRI) returned distances in Å for the entire protein rather than for the interface alone. This perspective could capture conformational changes outside the binding regions that would be missed by other measures. L_rms found 64–69% of all “ligands” (per definition the smaller of the two proteins in the interaction) not to exhibit conformational changes and to bind to the larger proteins at the same positions (RMSD <1.0 Å). Conversely, 10–14% of the interfaces differed very substantially between alternative experiments (>9.0 Å). Considering Face Position Similarity and L_rms at the same time suggested that about 1% of all comparisons did not differ by the first but differed substantially (>9 Å) by L_rms (Fig. S11 in Text S1). In other words, at least 1% of all the changes between different experiments can be attributed to conformational changes outside the binding region.

Another CAPRI measure, the I_rms, compared the shapes of the interface regions common to both protein pairs. We found these common regions to be very different in about 4% (e.g. due to a rotation of one of the proteins) and largely conserved in 80% (Fig. S9 in Text S1).

We confirmed the surprising result of interface variability without sequential changes through a variety of additional analyses. The degree to which interfaces were mostly robust (ratio between rightmost and leftmost bars in Fig. 3) was a function of the number of copies of a particular interaction in a complex (i.e., a function of the ‘interface copy number’; Methods; e.g. Fig. 1: S1/S3 observed once in C1): the more copies, the relatively lower the bars on the right and the higher on the left (Fig. S8 in Text S1). But all of this also varied between families and particular complex subgroups (Fig. S7 in Text S1). For instance, MHC (Major Histocompatibility Complex) interactions were much less diverse than others. In fact, they contributed importantly to our overall results, although they constituted only a small fraction of all interactions. Like many before us, we also had to choose key parameters to define an interface (Methods). As previous studies differed in these parameters, we also provided results for several alternative choices (Section S3.1 in Text S1). For instance, we included structures with a resolution >2.5 Å, used 4 Å instead of 6 Å as the minimal distance between two interacting residues or did not consider the change in solvent accessibility upon binding (dASA) when defining interface residues. These additional analyses demonstrated that some of our quantitative results depended crucially on the choice of critical parameters while the qualitative findings did not.

### Minor sequence variations slightly increased binding diversity (D-SameProt)

Two hetero-dimers can differ by minor sequence variations and still correspond to the same external interaction. Comparing these slightly different pairs (Fig. 3, D-SameProt) suggested considerably lower interface conservation than for the same pairs (Fig. 3, D-SameSeq): the most conserved bin (0.9–1.0) was reduced by about five percentage points for Face Position Similarity (Fig. 3A black vs. dark gray) and by nine percentage points for the L_rms measure (Fig. 3B black vs. dark gray). These reductions were evenly distributed over the other similarity ranges. This result can be cast into two opposing views. On the one hand, it suggested that a PPI network accurately reflected the interactions because different protein variants only had a small effect on interfaces. On the other hand, there *was* a significant influence of small sequence changes. For instance, the range of very different interfaces (0.0–0.5) by the Face Position Similarity measure rose from 12% to 17%. In other words, about one interface pair in six differed substantially.

### Conservation broke down when comparing interologous interfaces (D-Interolog)

When two experiments measured external interactions that did not correspond to the same protein pair, but to the same family pair (D-Interolog), interface conservation dropped significantly by both measures (Fig. 3, D-Interolog, rightmost bars; Face Position Similarity: 28–36%; L_rms: 7–11%). For Face Position Similarity, these differences largely originated from a shift toward intermediate levels of conservation, suggesting that most changes partially preserved the approximate interface location. The Sphere Radius Ratio supported this interpretation (Fig. S9 in Text S1). Nevertheless, the interfaces with clear dissimilarity also increased from 13% (D-SameSeq) to almost 30% (D-Interolog, Fig. 3, cumulative black to light gray bin with <0.5). This effect was stronger for L_rms: 33–40% of all comparisons were by this measure clearly dissimilar (>9 Å; Fig. 3[B], light gray vs. black). For these strong differences, the effects from conformational changes (Fig. S5 in Text S1) and from local interfaces appeared to act synergistically.

We hypothesized that families of interologs without alternative binding might have similar functions and that the same could be true for families with extreme binding diversity. Unfortunately, only for 18 Level Interolog clusters, interfaces were always maintained (Face Position Similarity >0.9 at 100%), while only 17 others always used very different interfaces (Face Position Similarity <0.5 at 100%). These numbers were too small to permit statistically significant analyses on the functional differences between those interactions. We still provided a list of those cases in Section S8 in Text S1. The two most extreme findings of this analysis were that the Gene Ontology (GO) [49] term “tetrapyrrole binding” appeared over-represented in the interactions that differed, while “cytoskeletal protein binding” appeared over-represented in the interactions that did not change.

### Trivially, removing alternative binding to the same homomer reduces diversity

With a special filter, we might remove all alternative binding of a protein to the same homomer from D-SameSeq to D-SameFam (Methods). Obviously, filtering out diversity will reduce the signal of diversity observed. Nevertheless, we performed this analysis. As expected, the observed effects dropped significantly (Table 1), most extremely for D-SameSeq, i.e. for the same pairs. The differential behavior between D-SameSeq and D-Interolog might be explained by sequence divergence increasingly leading to very different interfaces for the same protein pair and ultimately to different quaternary states. Despite all the filtering for homomers, varying interfaces remain frequent and still almost one third of the change seen in interologous pairs (D-Interolog) is also observed between the same pairs (D-SameSeq).

**Table 1:** Influence of homo-oligomerization.

	Original	Homomer filtered (sequence)	Homomer filtered (structure)
**Distribution**			
**D-SameSeq: 0.0–0.5**	11–16%	3–4%	-
**D-SameSeq: 0.9–1.0**	75–79%	84–88%	-
**D-SameProt: 0.0–0.5**	14–19%	4–7%	-
**D-SameProt: 0.9–1.0**	69–75%	80–84%	-
**D-Interolog: 0.0–0.5**	26–32%	11–16%	8–12%
**D-Interolog: 0.9–1.0**	29–35%	34–41%	38–47%

This table shows a summary of the Face Position Similarity distributions of Fig. 3 after excluding diversity introduced by a protein chain binding to the same homo-oligomer at different positions. We used two different definitions for homomers: at the sequence level, all chains in the assembly come from the same protein. In a ‘structural homomer’, they only come from the same family.

### Examples illustrate that interfaces can really differ substantially

**Figure 4:**
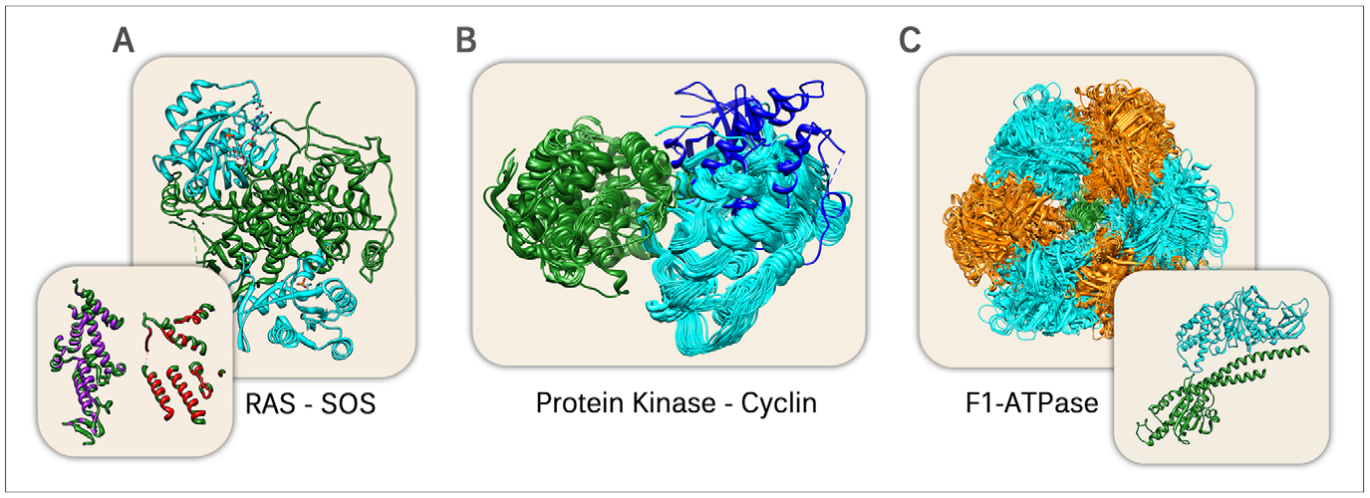
Three typical interactions exhibiting surprising variety. (**A**) Protein ‘ras’ binds to ‘son of sevenless’ (1NVV): alternative binding for sequence-identical pairs of proteins and without a multimeric context; the lower left panel shows the residues of the two interfaces in purple and red. (**B**) Natural dimeric interactions between proteins from the protein kinase and cyclin families (interface copy number 1; e.g. 1OI9). Cyclin chains (green) have been structurally aligned and superimposed. Protein kinases (cyan and blue) were subject to the same geometric translations. The blue chain has a recently discovered outlier interface (see text). (**C**) Superimposition of entire sequence-identical F1-ATPase complexes. Complexes were aligned and superimposed with the gamma chains (green). Alpha (orange) and beta (cyan) subunits were subject to the same geometric translations. In the main panel, we look at the complexes from the top. The inlet displays an interaction between a beta and a gamma subunit from the side.

Our finding that most interactions form identically when repeating the experiment might not be surprising. The observation that many interactions differed substantially, in contrast, appears much more counter-intuitive. Readers might attribute the difference to some mistake in the way we measure similarity or build our data sets. We addressed these concerns by expanding our analysis in many directions. On top, we analyzed ten case studies in more detail. Three are discussed in detail in the following (Fig. 4), the other seven in Section S7 in Text S1. Our entire collection of interesting protein pairs is available in Dataset S1.

#### The same proteins binding at entirely different interfaces

Our first example is the enzyme *ras* in complex with the nucleotide exchange factor SOS (*Son Of Sevenless*; Fig. 4A). *Ras* catalyzes the conversion of guanosin tri- to diphosphate (GTP→GDP). It needs the interaction with SOS in order to release GDP again after conversion. To this end, SOS provides a binding site that is highly specific for the ras-GDP complex. However, despite this specificity to ras-GDP, also SOS and ras-GTP can form a complex [50]. SOS has a second interface far away from the first that is specific for ras-GTP. It accelerates the reaction “ras-GDP→ras + GDP”. In other words, the ras-GTP-SOS complex separates GDP from ras faster than the uncomplexed SOS. Consequently, we have a positive allosteric modulation, in which both the active and the allosteric site are specific for exactly the same protein. This represents the rare case of sequence-identical protein pairs (D-SameSeq) binding very differently without prior homomerization of subunits (Table 1) and with a low interface copy number (2; Fig. S8 in Text S1). In Text S1, we discuss another similar case (Yersinia Pestis Antigen; Fig. S12A in Text S1).

#### Continuum of binding modes

Related work mostly differentiates alternative binding modes by clustering approaches. This implicitly suggests the assumption that the system could fall into alternative minima. Our results seem to support this assumption: interface similarities follow an extreme distribution, i.e. either they are very similar or very dissimilar (Fig. 3). When digging deeper, however, we easily found exceptions. We discuss one case in the following and two others in Fig. S12C,E in Text S1.

The dimeric interactions between cyclins and protein kinases (interface copy number 1) reveal a largely conserved binding cloud (Fig. 4B, green and cyan). Pairwise Face Position Similarities span a range from 0.6 to 1.0. An automated clustering of the structures would not accurately reflect the reality of this case because it would discriminate between the interfaces. Such a clustering might be improved by using the functions of the respective proteins in order to find correlations between interface and functional similarities. However, we found our current functional classification (GO) not to be comprehensive enough for this, yet (Section S3.2 in Text S1). Another complicating factor is that continua can not only be observed on the D-Interolog Level (as is the case here), but also on the Levels of D-SameProt and even of D-SameSeq (Fig. S12C,E in Text S1; namely hemoglobin and choleraholotoxin). For those two examples, GO-like functional annotation systems are trivially insufficient as they operate on the level of proteins, not protein variants or even interfaces.

Further extending the interface variability of cyclins and protein kinases is the recently discovered interface between cyclin D1 and protein kinase 4 (Fig. 4B, blue). Its discovery seems mainly due to improvements in experimental methods [51]. Hence, this might only be the beginning of an entirely new class of binding modes.

#### Structural homomers as a gate-way to higher-order complexation

Homomers can bind to another protein through alternative interfaces (Fig. S12D,F,E in Text S1). This concept can be generalized by introducing structural homomers, i.e. assemblies between proteins from the same family (Methods). The F1-ATPase structure (Fig. 4C) is such a case. Here, the alpha and beta subunit (Fig. 4[C]: orange and cyan) bind alternatingly to each other, thereby forming a hexameric ring with a central pore. The gamma subunit (Fig. 4C: green) winds through this pore with two long helices. This hexameric ring alone already represents two entirely different binding modes between the same protein pairs (D-SameSeq) and without homomerization of subunits. Two alpha subunits are always separated by a beta subunit and vice versa. Furthermore, all of these six chains bind to different positions on the gamma subunit, leading to two other interactions with great variability. Interface clouds of different structures, e.g. under varying conditions, reveal the dynamic nature of the interaction. Especially a rotation of the hexameric ring around the central helices is frequent.

The missing homo-oligomeric context is hidden: the hexameric ring appears to be a homomer. Subunits alpha and beta have a RMSD of 1.3 Å. Only the sequences and eventually their original proteins, reveal that we are actually dealing with heteromers. However, both of the proteins come from the same family, so that we have a case of a ‘structural homomer’, i.e. an assembly that appears to be homomeric, but has actually undergone significant sequence divergence. Similar to traditional homomers, the alternative binding to another protein (subunit gamma) is evident from the structure within seconds and homomerization might be essential (i.e. interactions between alpha and gamma subunits might not be stable without the hexameric ring). Filtering alternative binding to the same structural homomer (Methods), the only remaining variability is the rotation around the two central helices of subunit gamma. This is a good example why homomeric filtering might come short from a biological point of view: What if subunits alpha and beta would not have the same family? Would it make the variability between alpha and gamma subunits any more or less biologically relevant? Unfortunately, current data does not lead to specific answers, yet. Similar examples for structural homomers are some hemoglobin structures (Fig. S11 in Text S1) and the exosome complex by Lorentzen et al. [52] (2JE6).

#### Other interesting cases

We had to exclude other interesting examples to limit the length of this work. These included the D-SameProt comparisons of two nitrogenase complexes (2AFH, 1QH1) exhibiting alternating quaternary states [53]; two amine dehydrogenase complexes (2J57, 2IUP) with different enzymatic activities (Section S3.2 in Text S1); dual binding modes of cohesion and dockerin (2CCL, 1OHZ); and two interologous interactions for which the according Swiss-Prot sequences are identical and only differ in their organisms (2PE6, 2VRR; dimeric). Notable previous publications reporting different binding modes include, e.g., [54] (histidine kinases; 1U0S) and [55] (cytochrome C; 2B11).

#### Summary of analyzing examples

While our examples confirmed the overall trends, they also suggested that the averages above reveal only the tip of the iceberg: if there is one reasonable measure for interface similarity by which two experimental solutions differ, then this observation suggests variability. To complicate matters further, we observe “rotational interfaces” (F1-ATPase) and see that ligand binding can have great impact on interface specificity (ras-SOS). On another note, our tests with alternative data set parameters, e.g. changing structural resolution, revealed that the variability that we see is not explained by experimental or procedural inaccuracies. Thus, the PDB structures clearly tell a tale of unexpected variability and dynamics of biological interactions.

## Discussion

### How can proteins interact differently?

Empirically, we found several reasons for the same two proteins to have different interfaces (D-SameSeq). The simplest was merely technical: some experimental findings may not have been completely correct. We reduced this effect by excluding complexes with resolutions >2.5 Å, but even structures at 1.2 Å can contain errors [56], [57]. Another reason was local flexibility or disorder: many proteins have local regions that are natively unstructured and these often form protein-protein interfaces [58]; such regions are difficult to track experimentally. Often, the N- and C-termini contributed to the observed interface variability. Another reason was environmental differences: despite all efforts, we could not entirely exclude artificial interfaces due to crystal packing. Different pH values could trigger conformational changes, as was the case for small ligands or other interaction partners. The presence of another protein changing the overall structure of a complex played a similar role. In all that, however, we still miss one important aspect: proteins often have evolved to interact in different ways. For such cases of biologically important alternatives, we might interpret the variety observed in a single PDB structure as an example of one protein binding to multiple copies of the same interaction partner.

There were various reasons why variability in binding was higher between sequentially modified proteins than for identical proteins. The modifications that preserved the original protein (D-SameProt) were usually point mutations (i.e. changes of single amino acids, e.g. by site directed mutagenesis or in the form of Nucleotide Polymorphisms [SNPs]). Others included protein tags at the N- or C-terminus (e.g. to facilitate protein purification), post-translational modifications (protein cleavage) and alternative splicing. For interologs (D-Interolog), finally, there was also evolutionary driven sequence divergence. As described before, however, the mere presence of insertions or deletions was not enough for low interface similarity: we reduced structures to common residues before comparing them. Thus, the increase in variability was actually the result of changes in the common parts of two structures.

### Continuum of binding modes rather than major clusters?

Using similar measures as we did, other groups [33], [37] have found that many families interact in more than one way. Our analyses support this result. However, they also reveal that the differences in interfaces span the entire spectrum of the distribution, especially for D-Interolog. Only 18 of the 151 pairs of families completely conserved the binding modes. This finding suggests the model of a continuum of binding modes rather than clearly defined groupings, e.g. as obtained by clustering at predefined thresholds. Furthermore, in our results, about one third of the variability observed in a family-family interaction appeared to be protein-intrinsic in the sense that it was also observed between sequence-identical pairs (D-SameSeq), i.e. did not originate from sequence variations (as, e.g. for D-Interolog).

### Is variability caused by homomers a special case?

As mentioned before, alternative interfaces might be due to the intrinsic capability of proteins to bind at different positions. This is often encountered among homo-oligomers [59]. In our case, however, it leads to a debatable scenario: a protein A can bind to multiple copies of protein B, all of which alone form a homo-oligomeric complex (Fig. 2). Do we then have to treat the various external interfaces between the same proteins as one interface, or are they indeed individual interfaces that ought to be differentiated? We argue for the second case: first, considering the homomer a requirement for the hetero-interactions implies that by disabling the homomerization (e.g. through site-directed mutagenesis), we also lose the interaction. This is not always the case [60]. Secondly, it is unclear why such a filtering should be limited to homo-complexes. Also the formation of a *hetero*-multimer could be a requirement for the interaction with another protein. Studying which interactions remain after disabling the potentially highly complex hetero-multimer is much beyond the currently available data. Finally, also the original publication of a complex usually describes different interfaces to the same homomer as separate interfaces.

### Conclusions

Our results raise the question whether the molecular details of protein-protein interactions (PPIs) are crucial for function. Protein crystallography captures static views on those molecular details along with some information about the dynamic nature of PPIs. If the details always had to be the same to guarantee function, different experiments would identify the same interfaces. We applied many reasonable ways of measuring interface similarity in order to analyze the consistency of the molecular details of protein-protein interactions between different experiments. For sequence-identical pairs of proteins, i.e. the same biological interaction, most interfaces were almost completely conserved by all measures. However, all measures also revealed an unexpected variety. Depending on how much detail we required to be similar in order to consider two experiments to yield the same results, we found 11–37% of all observations to have significant differences, and up to 10% to be completely different. One important result was that this was a significant fraction of the difference observed between homologous PPIs. Put differently, over a third of the differences in the interactions between pairs of homologous proteins are also observed between identical proteins. These numbers may challenge the notion that the maintenance of the molecular details is crucial for function. At least, our results suggest that there appear to be many alternative solutions to maintain or actually enable the intricate molecular details: change seems an extremely frequent exception for protein-protein interfaces.

## Supporting Information

Dataset S1 In this file, we list all pairs of hetero-dimers leading to a Face Position Similarity below 0.9. Additional columns provide the copy numbers, L_rms, an indicator for the best homomer match and the type of sequence divergence.
(ZIP)

Text S1 In this text, we give details about various methods and analyses found in the main manuscript and provide additional results.
(PDF)
