# Supplementary material for: Alternative Protein-Protein Interfaces Are Frequent Exceptions
Source: PLoS Comput Biol. 2012 Aug 2;8(8):e1002623. doi: 10.1371/journal.pcbi.1002623 (PMC3410849; doi:10.1371/journal.pcbi.1002623)
Supplement: Text S1 — In this text, we give details about various methods and analyses found in the main manuscript and provide additional results. (PDF) [file pcbi.1002623.s002.pdf]

Alternative Protein-Protein Interfaces Are Frequent Exceptions

Supplementary Online Materials

Tobias Hamp and Burkhard Rost

Contents

S1 Calculation of Interface Similarity Distributions **3**
S1.1 Clustering Procedure 3
S1.2 Detailed Definitions of Similarity Distribution 4
S1.3 Standard Errors 5
S1.4 Cross-correlating Distributions 6

S2 Similarity Measures **6**
S2.1 Face Position Similarity 8
S2.2 Interface Position Similarity 8
S2.3 Sphere Radius Ratio 8
S2.4 Convex Hull Overlap 9
S2.5 Interface Composition Similarity 9
S2.6 Domain Number Ratio 9
S2.7 Family Interaction Similarity 10
S2.8 RMSD 10
S2.9 L_rms 10
S2.10 I_rms 10
S2.11 Comparison of Measures 11

S3 Data Set Analysis **12**
S3.1 Influence of Data Set Parameters 12
S3.2 PPI Data Set Properties 13
S3.3 On the Accuracy of PISA 17

S4 Significant Complex Subgroups **19**

S5 Complex Size vs. Interface Variation **21**

S6 Distributions of Interface Similarities **22**

S6.1 Face Similarity Distributions 22
S6.2 Interface and Domain Similarity Distributions 24
S6.3 Cross-correlations 24

S7 Additional Sample Structures 27

S8 Functions of Families With and Without Interface Variability 28

S1 Calculation of Interface Similarity Distributions

In the following, we give a formal definition of our clustering procedure and the calculation of (inter-)face similarity distributions across different clustering Levels and types of sequence divergence. Less detailed description are given in the Methods. We explain interface similarity measures in the Methods and Section S2.

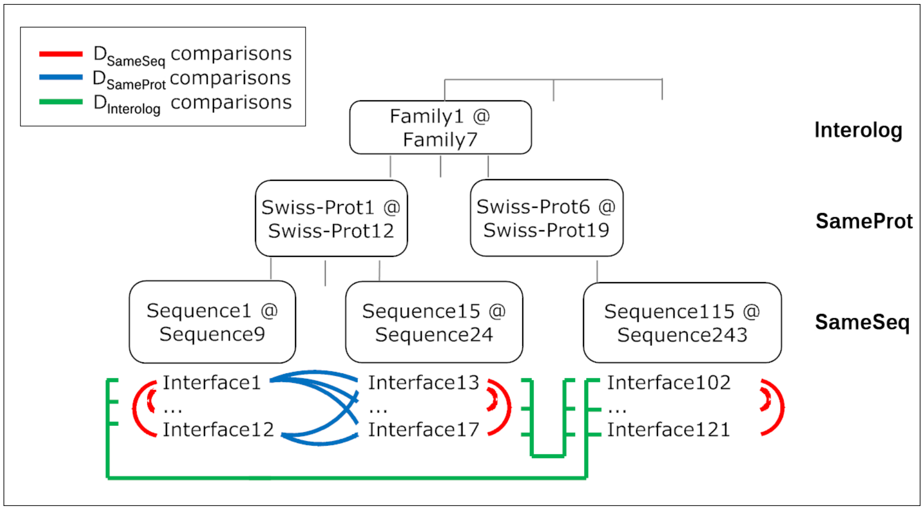

Figure S1: Clustering procedure and calculation of similarity distributions. We clustered interfaces over 3 different levels. In the first level, 'SameSeq', interfaces coming from identical sequence pairs were grouped together. Level SameSeq clusters from the same Swiss-Prot pair ('protein pair') were then merged in the same Level SameProt cluster. Level SameProt clusters were grouped together into the same Level Interolog clusters if they came from the same pair of Pfam [8] families. We used this clustering to derive different interface similarity distributions, based on three different types of interface comparisons: First, we compared only interfaces from the same pair of sequences (red; distribution $D_{SameSeq}$), then only those from the same pair of proteins (blue; distribution $D_{SameProt}$) and finally we only required them to come from the same family pair (green; distribution $D_{Interolog}$)

S1.1 Clustering Procedure

In case of the PDB, only grouping our interactions by sequence pairs would result in some highly overrepresented protein families that would dominate any interface similarity distribution. Thus, we need to cluster interfaces further and then to respect this clustering during the calculation of the distributions. We

hierarchically cluster hetero-dimers over three levels, corresponding to increasing levels of sequence divergence.

Let A be a hetero-dimer from our data set. It has two chains from two different proteins X and Y. We denote the two chains as C_X^A and C_Y^A. Let further $seqres(C_X^A)$ be the SEQRES sequence, $sp(seqres(C_X^A))$ the Swiss-Prot sequence and $pfam(sp(seqres(C_X^A)))$ the Pfam families of chain C_X^A. Let those sequences and families be defined analogously for the other chain C_X^B.

On the first clustering Level (SameSeq), we assgin two hetero-dimers A and B (chains $C_{X'}^B$ and $C_{Y'}^B$) to the same cluster if they have the same pair of SEQRES sequences, i.e. $seqres(C_X^A) = seqres(C_{X'}^B)$ and $seqres(C_Y^A) = seqres(C_{Y'}^B)$. Consequently, we can represent a Level SameSeq cluster by a pair of SEQRES sequences $(seqres_i, seqres_j)$, because all the hetero-dimers in a cluster have exactly the same SEQRES sequence pair.

On the second clustering level (SameProt), each cluster consists of several SameSeq clusters. We merge two Level SameSeq clusters $(seqres_i, seqres_j)$ and $(seqres_{i'}, seqres_{j'})$ if they point to the same pair of Swiss-Prot entries, i.e. $sp(seqres_i) = sp(seqres_{i'})$ and $sp(seqres_j) = sp(seqres_{j'})$. We denote a SameProt cluster by its pair of Swiss-Prot entries (sp_k, sp_l).

In the third clustering Level (Interolog), finally, two Level SameProt clusters (sp_k, sp_l) and $(sp_{k'}, sp_{l'})$ are grouped together if they have the same Pfam family composition, i.e. $pfam(sp_k) = pfam(sp_{k'})$ and $pfam(sp_l) = pfam(sp_{l'})$.

S1.2 Detailed Definitions of Similarity Distribution

In the following, we describe the calculation of interface similarity distributions. Mathematically, they fall into the category of so-called 'finite mixture distributions', i.e. weighted averages over many uncorrelated individual distributions. We reduce the influence of the redundancy found in the PDB by giving the same weight not only to sequence pairs, but also to protein pairs and eventually family pairs.

S1.2.1 Distribution $D_{SameSeq}$

Here, we describe how we can use the clustering to derive non-redundant interface similarity distributions. Let $Cl_x \in \{Cl_1, \ldots, Cl_s\}$ be the set of Level SameProt clusters in Level SameFam cluster x, $Cl_{x,y} \in \{Cl_{x,1}, \ldots, Cl_{x,t}\}$ the set of Level SameSeq clusters in Level SameProt cluster (x, y) and $EI_{x,y,z}^1, \ldots, EI_{x,y,z}^v$ the external interfaces of Level SameSeq cluster (x, y, z). We first calculate the set of pairwise similarities $S_{x,y,z} = \{sim(EI_{x,y,z}^i, EI_{x,y,z}^j) \mid i \neq j \wedge i, j \in \{1, \ldots, v\}\}$ where $sim(EI_{x,y,z}^i, EI_{x,y,z}^j) \in [0, 1]$ was the result of one of the similarity measures as described in the Methods and Section S2 and $EI_{x,y,z}^i$ always came from a different PDB entry than $EI_{x,y,z}^j$. We then defined a discrete probability distribution $P_{x,y,z}(X \in [a_k, a_{k+1}]) = \frac{|\{s | s \in [a_k, a_{k+1}] \wedge s \in S_{x,y,z}\}|}{|\{S_{x,y,z}\}|}$ with $a_k = \frac{k}{n}, k \in (0, 1, \ldots, n-1)$ and n typically set to 10, which gave the chance of the interface similarity lying between a_k and a_{k+1} after randomly

picking two structures corresponding to the pair of protein sequences given by $Cl_{x,y,z}$ (Note that this is essentially a maximum likelihood estimation for the unknown parameters p of typically $n = 10$ different Bernoulli-distributed random variables). Repeating this procedure for all Level SameSeq clusters in $Cl_{x,y}$ leads to the i.i.d set $\{P_{x,y,1}, \ldots, P_{x,y,|Cl_{x,y}|}\}$ and subsequently to the Level SameProt similarity distribution $P_{x,y}(X \in [a_k, a_{k+1}]) = \frac{1}{|Cl_{x,y}|}[P_{x,y,1}(X \in [a_k, a_{k+1}]) + \cdots + P_{x,y,|Cl_{x,y}|}(X \in [a_k, a_{k+1}])]$. We obtain P_x in the same way as $P_{x,y}$, i.e.: $P_x(X \in [a_k, a_{k+1}]) = \frac{1}{|Cl_x|}[P_{x,1}(X \in [a_k, a_{k+1}]) + \cdots + P_{x,|Cl_x|}(X \in [a_k, a_{k+1}])]$. Finally we define the overall distribution $D_{SameSeq}$ as: $D_{SameSeq}(X \in [a_k, a_{k+1}]) = \frac{1}{s}[P_1(X \in [a_k, a_{k+1}]) + \cdots + P_s(X \in [a_k, a_{k+1}])]$.

S1.2.2 Distribution $D_{SameProt}$

Now, we compare interfaces coming from different Level SameSeq clusters. Given two Level SameSeq clusters $Cl_{x,y,z}$ and $Cl_{x,y,z'}$ with interfaces $EI^1_{x,y,z}, \ldots, EI^v_{x,y,z}$ and $EI^1_{x,y,z'}, \ldots, EI^{v'}_{x,y,z'}$, we define the pairwise similarities as $S_{x,y,(z,z')} = \{sim(EI^i_{x,y,z}, EI^j_{x,y,z'}) \mid i \in \{1, \ldots, v\} \wedge j \in \{1, \ldots, v'\}\}$. The corresponding distribution is then defined as $P'_{x,y,(z,z')}(X \in [a_k, a_{k+1}]) = \frac{|\{s|s \in [a_k, a_{k+1}] \wedge s \in S_{x,y,(z,z')}\}|}{|\{S_{x,y,(z,z')}\}|}$ with $a_k = \frac{k}{n}, k \in (0, 1, \ldots, n-1)$. Consequently, we can calculate the Level SameProt distribution $P'_{x,y}(X \in [a_k, a_{k+1}]) = \frac{1}{|Cl_{x,y}|(|Cl_{x,y}|-1)}[P_{x,y,(1,2)}(X \in [a_k, a_{k+1}]) + \cdots + P_{x,y,(1,|Cl_{x,y}|)}(X \in [a_k, a_{k+1}]) + \cdots + P_{x,y,(|Cl_{x,y}|,|Cl_{x,y}|-1)}(X \in [a_k, a_{k+1}])]$. Substituting P with P', we obtain P'_x in the same way as P_x in $D_{SameSeq}$, and ultimately also the overall distribution $D_{SameProt}$ in the same way as $D_{SameSeq}$.

S1.2.3 Distribution $D_{Interolog}$

Finally, we compare interfaces coming from the same Level Interolog cluster x, but different Level SameProt clusters (x, y) and (x, y'). To this end, we define a Level SameProt similarity distribution $P''_{x,(y,y')}(X \in [a_k, a_{k+1}]) = \frac{1}{|Cl_{x,y}||Cl_{x,y'}|}[P''_{x,(y,1),(y',1)}(X \in [a_k, a_{k+1}]) + \cdots + P''_{x,(y,1),(y',|Cl_{x,y'}|)}(X \in [a_k, a_{k+1}]) + \cdots + P''_{x,(y,|Cl_{x,y}|),(y',|Cl_{x,y'}|)}(X \in [a_k, a_{k+1}])]$ where $P''_{x,(y,z),(y',z')}(X \in [a_k, a_{k+1}]) = \frac{|\{s|s \in [a_k, a_{k+1}] \wedge s \in S_{x,(y,z),(y',z')}\}|}{|\{S_{x,(y,z),(y',z')}\}|}$ with $a_k = \frac{k}{n}, k \in (0, 1, \ldots, n-1)$ and $S_{x,(y,z),(y',z')} = \{sim(EI^i_{x,y,z}, EI^j_{x,y',z'}) \mid i \in \{1, \ldots, |Cl_{x,y,z}|\} \wedge j \in \{1, \ldots, |Cl_{x,y',z'}|\}\}$. Before, we used the set of distributions defined as $P_{x,y}$ in order to calculate P_x. We now replace this set with the new newly derived $P''_{x,(y,y')}$ distributions and obtain P''_x. Performing analogous steps for the overall distribution, we calculate $D_{Interolog}$.

S1.3 Standard Errors

We calculated standard errors of distributions $D_{SameSeq}$ to $D_{Interolog}$ with a multi-level bootstrapping approach. From the n Interolog clusters which con-

tributed to a D distribution, we first re-sampled with replacement until we had a new list of n Interolog clusters (in which some entries might have been duplicates). In other words, we bootstrapped the Interolog clusters. For each Interolog cluster in this bootstrap, we then bootstrapped its SameProt clusters. For each of the SameProt clusters in this sub-bootstrap, we bootstrapped the SameSeq clusters and finally the interface similarities in each bootsrapped SameSeq cluster. Thus, in the end, our overall boostrap was a resampling over all Levels and clusters. Next, we re-calculated the D distributions with this bootstrap and saved it. Then, we repeated all of the above 200 times.

Ultimately, we had 200 different estimates for each of the 10 bins in a D distribution. The standard error being defined as the standard deviation of a target statistic and the occurrence of a bin being this target statistic, the standard deviation of a bin over those 200 estimates defined its standard error. Note that this procedure is independent of the number of bins: for example, if we are interested in the occurrence of the range 0.0 to 0.5, we only have to change the n parameter for the D distribution to 2 (creating two bins: 0.0 to 0.5 and 0.5 to 1.0) and repeat all of the above.

S1.4 Cross-correlating Distributions

In order to capture interface differences with two measures simultaneously, we compiled so-called '2D distributions'. In this context, the function $sim(EI^i, EI^j)$ of $D_{SameSeq}$ no longer returned a single number, but a triple, with both elements coming from different similarity measures. Subsequently, we redefined:

$$P_{x,y,z}(X \in ([a_k, a_{k+1}], [a_l, a_{l+1}])) = \frac{|\{(s_1,s_2)|s_1 \in [a_k,a_{k+1}] \wedge s_2 \in [a_l,a_{l+1}] \wedge (s_1,s_2) \in S_{x,y,z}\}|}{|\{S_{x,y,z}\}|}$$

with

$a_k = \frac{k}{n}, a_l = \frac{l}{n}, k, l \in (0, 1, ..., n-1)$ and n typically set to 10.

S2 Similarity Measures

In the following, we compare the interface between chains C_X^A and C_Y^A in heterodimeric structure A with the interface between chains C_X^B and C_Y^B in heterodimeric structure B. X and Y indicate two different proteins (SameSeq, SameProt) or families (Interolog). The atomic sequences of C_X^A and C_X^B are related, but not necessarily the same. In particular, parts of C_X^A might be missing in C_X^B and vice versa because of, e.g., experimental inaccuracies or evolutionary insertions and deletions (analogous statements for C_Y^A and C_Y^B). Those cases should not lead to low interface similarities as they should only be reported for actual binding mode changes. Consequently, we reduced A and B to common residues before comparing their interfaces. We found common residues in the following ways. (In case you want to skip the details of this procedure, simply assume that corresponding chains had the same number of residues with a 1:1 mapping between them and continue with Section S2.1)

Let $atomseq_X^A$ be the atomic sequence, $seqres_X^A$ the SEQRES sequence and sp_X^A the Swiss-Prot sequence of chain C_X^A. Let the same sequences be defined analogously for the other chains C_X^B, C_Y^A and C_Y^B.

If corresponding chains had the same SEQRES sequence (i.e. $seqres_X^A = seqres_X^B$), both of their atomic sequences $atomseq_X^A$ and $atomseq_X^B$ were semi-globally aligned to $seqres_X^A$ (BLOSUM62 as alignment matrix). Common residues could then be identified by identical positions in this 3-sequence alignment: all columns without gaps corresponded to common residues, all other residues were discarded. Analogous statements hold again for chains Y, but we will restrict ourselves to the case of X here and in the following for reasons of simplicity.

In case corresponding chains had different SEQRES sequences, but were variants of the same protein (i.e. mapped to the same Swiss-Prot entry), the atomic sequences $atomseq_X^A$ and $atomseq_X^B$ were first semi-globally aligned to the SEQRES sequences $seqres_X^A$ and $seqres_X^B$, respectively. Then we aligned both $seqres_X^A$ and $seqres_X^B$ to the representative Swiss-Prot sequence sp_X^A (Note that $sp_X^A = sp_X^B$ because C_X^A and C_X^B come from the same Swiss-Prot entry). In case we had to introduce gaps in $seqres_X^A$ during the alignment to sp_X^A, they were also added at the same positions in the $atomseq_X^A$ alignment, thus preserving each alignment and its length (analogous steps for B). In the end, common residues of $atomseq_X^A$ and $atomseq_X^B$ mapped to the same position in the Swiss-Prot sequence and were identified by gapless columns in the final 5-sequence alignment.

If the protein chains C_X^A and C_X^B came from different Swiss-Prot entries but the same family, the atomic residues were aligned to Swiss-Prot positions as before, i.e. $atomseq_X^A$ was aligned to $seqres_X^A$ and $seqres_X^A$ was aligned to sp_X^A, with analogous steps for B. Finally, we aligned sp_X^A and sp_X^B. In case of gaps in the alignment between sp_X^A and sp_X^B, they were added at according positions in both the atomic and SEQRES sequence alignments. In the final 6-sequence alignment, atomic residues mapping to the same position in this last alignment were considered common residues and could again be identified by columns without a gap in each of the 6 rows.

Note that structure alignments were not used for two reasons. Firstly, we wanted to safely identify missing residues. Using SEQRES and Swiss-Prot sequences somewhat improves the sensitivity in this context. Consider for example the case of C_X^A and C_X^B coming from different parts of the same gene (as it might happen for example after post-translational cleavage). A structure alignment might align C_X^A and C_X^B, find common residues and hence allow the comparison of their interfaces. The above procedure, on the other hand, correctly suggests that the two proteins have no common background and thus have to be excluded from the comparison. Secondly, we would have had to align structures before any pairwise interface comparison because the atomic sequences often differ slightly. This was unfeasible. Following the above procedure, we only had to align the atomic sequence to the SEQRES sequence once and could then infer common residues via this alignment.

S2.1 Face Position Similarity

Face Position Similarity first creates four different residue sets $F_X^A, F_Y^A, F_X^B, F_Y^B \subset \mathbb{N}$ by determining the position of each interacting amino acid on X and Y for both A and B and then peforms comparisons between F_X^A and F_X^B and between F_Y^A and F_Y^B via s_{fps}:

$$\begin{aligned} s_{fps} &: 2^{\mathbb{X}} \times 2^{\mathbb{X}} \to [0,1] \\ (F_1, F_2) &\mapsto \frac{|F_1 \cap F_2|}{\sqrt{|F_1||F_2|}} \end{aligned} \tag{1}$$

where $\mathbb{X} = \mathbb{N}$ and F_1 and F_2 are two sets of face residue positions so that each of their elements points to exactly one amino acid on a common protein sequence. For simple single distributions (Section S1.2 and Methods), the two similarities were subsequently arithmetically averaged. In the 2D plot (Section S1.4 and Methods), they were treated separately so that only corresponding faces were compared.

S2.2 Interface Position Similarity

Interface Position Similarity captures similarity analogously to Face Position Similarity, but also includes information about which particular pairs of amino acids interact. A and B are projected onto two sets $IF_A, IF_B \subset \mathbb{N} \times \mathbb{N}$ which both contain tuples of positions of interacting residues. An element $(i,j) \in IF_A$ for example indicates that in structure A, the i-th residue of X has contact with the j-th residue of Y. The similarity between IF_A and IF_B is calculated via s_{fps} with $\mathbb{X} = \mathbb{N} \times \mathbb{N}$ (see Eq. 1).

S2.3 Sphere Radius Ratio

Sphere Radius Ratio first uses the residue position sets $F_X^A, F_X^B \subset \mathbb{N}$ as defined in Section S2.1 to specify two different face locations on chain C_X^A and stores the coordinates of the corresponding atoms in sets $K_A^{C_X^A}, K_B^{C_X^A} \subset \mathbb{R}^3$ (note that only one of the two sets contains atoms which are actually interacting in structure A). Their similarity is then calculated via s_{srr}

$$\begin{aligned} s_{srr} &: 2^{\mathbb{R}^3} \times 2^{\mathbb{R}^3} \to [0,1] \\ (K_1, K_2) &\mapsto \frac{r_{mbs}(K_1)}{r_{mbs}(K_1 \cup K_2)} \end{aligned} \tag{2}$$

where $r_{mbs} : 2^{\mathbb{R}^3} \to \mathbb{R}$ returns the radius of the smallest sphere which encompasses all of the given coordinates.

Applying this procedure not only to C_X^A, but also to C_Y^A,C_X^B and C_Y^B, one obtains all in all four distinct similarities $s^{A_X}, s^{A_Y}, s^{B_X}$ and s^{B_Y}. As differences between similarities corresponding to the same chain but different structures (e.g. s^{A_X} and s^{B_X}) should mainly be attributed to backbone flexibilities, we

always averaged those arithmetically, reducing the number of similarities for one pair of external interfaces to two. Analogously to Face Position Similarity, these were then again averaged when deriving 1D distributions (Section S1.2).

S2.4 Convex Hull Overlap

Using $K_A^{C_X^A}$ and $K_B^{C_X^A} \subset \mathbb{R}^3$ as defined above, Convex Hull Overlap first calculates their convex hulls $H_A^{C_X^A}$ and $H_B^{C_X^A}$ and then defines s_{cho} as:

$$\begin{aligned} s_{cho} : \mathbb{P}_3 \times \mathbb{P}_3 &\rightarrow [0, 1] \\ (H_1, H_2) &\mapsto \frac{vol(H_1 \cap H_2)}{max(vol(H_1), vol(H_2))} \end{aligned} \tag{3}$$

where $\mathbb{P}_3$ is the space of polyhedra in $\mathbb{R}^3$, $\cap : \mathbb{P}_3 \times \mathbb{P}_3 \rightarrow \mathbb{P}_3$ a function which determines the intersection of two polyhedra and $vol : \mathbb{P}_3 \rightarrow \mathbb{R}$ a function which returns the volume of a polyhedron.

Analogously to Sphere Radius Ratio, creating the convex hulls and calculating s_{cho} not only for C_X^A, but also for C_Y^A,C_X^B and C_Y^B, one obtains four distinct similariy values for each pair of external interfaces. These were then averaged as in the previous Section. Convex Hulls were calculated with the QHull package [1].

S2.5 Interface Composition Similarity

For both sets, $F_A = F_X^A \cup F_Y^A$ and $F_B = F_X^B \cup F_Y^B$ (Section S2.1), Interface Composition Similarity replaces each residue position $i \in F_A, F_B$ with the corresponding amino acid $aa_i \in \Sigma$, where Σ is the set of 20 amino acids. An interface can then be represented as a function $c_X : \Sigma \rightarrow \mathbb{N}$ giving the number of occurrences of each amino acid in an interface X. To compare interface compositions we define s_{aac} similarly to s_{fps}:

$$\begin{aligned} s_{aac} : C \times C &\rightarrow [0, 1] \\ (c_1, c_2) &\mapsto \frac{\sum_{\sigma \in \Sigma} |c_1(\sigma) - c_2(\sigma)|}{\sqrt{\sum_{\sigma \in \Sigma} c_1(\sigma) * \sum_{\sigma \in \Sigma} c(\sigma)}} \end{aligned} \tag{4}$$

where C is the space of interface functions c_X.

S2.6 Domain Number Ratio

We mapped each SCOP and CATH domain onto our sequences and counted the number of domains involved in the interaction for each external interface. A domain was involved if it contributed at least one binding residue to the interface. For each fully annotated pair of interfaces, we defined Domain Number Ratio as the ratio between the smaller and the larger of the two domain numbers.

S2.7 Family Interaction Similarity

The interface of an external interaction not only corresponds to pairs of interacting residues as defined by Interface Position Similarity, but also to pairs of interacting protein families. Thus, we employed SCOP and the domain mapping of Domain Number Difference to study to what degree pairs of interacting families change when comparing two external interfaces. For each structure, we compiled the set of all family pairings, analogously to IF_A and IF_B of Interface Position Similarity (S2.2). The actual similarity could then be derived by s_{fps} with $\mathbb{X} = \mathbb{F} \times \mathbb{F}$, where $\mathbb{F}$ was the space of SCOP families .

S2.8 RMSD

Actually not a similarity measure for interfaces, but rather for pairs of protein chains, we calculated the Root Mean Square Deviation (RMSD) of two binary heterocomplexes. To this end, we first separately superimposed C_X^A and C_X^B and then C_Y^A and C_Y^B. The average RMSD of both superpositions then represented the final similarity value which could be processed like any other similarity described before.

S2.9 L_rms

In order to link our results to related work, we introduce the measure L_rms from the CAPRI experiments [6]. Here, we first superimpose the bigger of the two chain pairs (i.e. either C_X^A and C_X^B or C_Y^A and C_Y^B). Then, we apply the transformation of the superposition to the remaining chains and calculate their RMSD. This measures how far the two smaller chains are apart in space. Note that L_rms is an interface *distance* measure which does not return a value between 0.0 and 1.0, but a distance in Å.

S2.10 I_rms

The I_rms is again a measure of the CAPRI experiments [6]. First, we redefine the interface between C_X^A and C_Y^A: Now, every pair of residues with at least one atom pair closer than 10Å is part of the interface (before, it was 6Å). Then, we determine equivalent *interface* residues of A and B: we first create two sets of residue positions: $R_{commonIF}^X = F_X^A \cap F_X^B$ and $R_{commonIF}^Y = F_Y^A \cap F_Y^B$ (see S2.1 for definitions of the F sets). Then we reduce C_X^A and C_X^B to the residues in $R_{commonIF}^X$, with analogous steps for Y. In these new interface structures, we remove all non-backbone atoms.

This leaves us with two new structures which have the same amount of atoms. All these atoms are both part of the interface and the protein backbone. Next, we optimally superimpose the structures and calculate the RMSD. This RMSD is the I_rms. In case we could not find common interface residues before, we returned maximum RMSD.

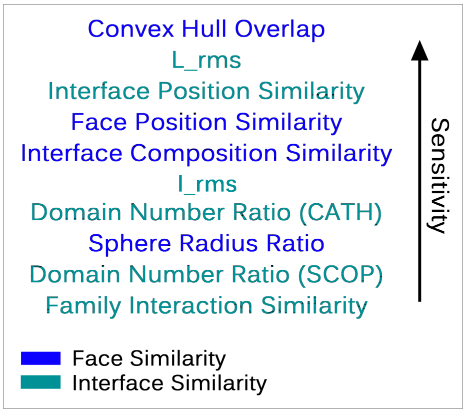

Figure S2: A hierarchy of interface similarity measures. We have looked at the 0.9 to 1.0 bins of the $D_{SameSeq}$ distributions and used them to compile a hierarchy of measures based on sensitivity. We differentiate between measures that look at both sides of the interface at the same time or incorporate interfacial contacts (Interface Similarity) and those which first treat each side of the interaction separately and then average their conservations (Face Similarity).

S2.11 Comparison of Measures

We have introduced a number of measures which capture different aspects of interface similarity. If they find dissimilarity, they assign lower values with different amplitudes, what makes them essentially incomparable in absolute terms. We can, however, compile a hierarchy of which measure sees differences more often than others based on the empirical results presented throughout the manuscript. We show it in Fig. S2. Here, we see that Convex Hull Overlap is the overall most sensitive face similarity measure. Only closely behind in terms of sensitivity follows L_rms, which is, however, strongly influenced by backbone movements of the entire proteins. The Face and Interface Position Similarity measures, coming next in the list, are both exclusively residue based. Interface Position Similarlity takes into account conservartion of residue-residue contacts and is therefore more susceptible to change. The I_rms, on one hand, is in principle quite sensitive because we chose very fine grained RMSD thresholds (steps of 0.5Å). One the other hand, it misses dissimilarities due to the reduction to common interface residues and also similarities if no such residues could be found. The Interface Composition Similarity (residue based) and Sphere Radius Ratio (atom based) already fall into the class of rather robust measures. As a specialty, both almost never assign low similarities. In case of the Interface Composition Similarity, this is mainly due to random effects. Two interfaces usually have similar dimensions which is why Sphere Radius Ratio always sees some similarity. Finally, we have the domain based similarity measures. CATH domain assignments change slightly more often between interfaces than SCOP assignments. Obviously, entire SCOP domain families are even more conserved

than domains.

S3 Data Set Analysis

S3.1 Influence of Data Set Parameters

In related publications, there are usually a number of ad-hoc decision when selecting structures and defining interfaces. To exclude experimental or sampling bias in this context, we investigated the influence of some of the arguably most crucial alternatives: including structures with a resolution above 2.5Å; setting the minimal distance of two residues to be considered interacting to 4Å instead of 6Å; using ΔASA (change of accessible surface area) as a means to correct for interacting residues slightly above a given distance cutoff; using level SameSeq clusters with less than 5 members instead of 5 or more members.

While the first two points can be commonly encountered, the use of ΔASA when defining interfaces (as opposed to faces) was mainly an effort to retain consistency with the PISA service, which exclusively uses ΔASA to define face residues. The use of clusters with less than 5 members finally was a test in how far cluster size influences the expected similarity of two interfaces.

We interpreted the options above as parameters which can adopt two distinct values, on and off, and used one of our most sensitive similarity measures, Interface Position Similarity (Section S2.2), to assess their effect. For a particular parameter value combination, we first calculated the discrete probability distribution $D_{SameSeq}$ (Section S1.2 and Methods). Put simply, this corresponds to the average distribution of pairwise interface similarities for identical protein pairs in a redundancy reduced version of the PDB. This procedure was repeated for all possible value combinations, resulting in $2^4 = 16$ different distributions. One could then create 8 pairs of distributions for each parameter p_i by only changing the value of p_i and keeping the other parameters fixed. For example, there were 8 combination with low-resolution structures included and 8 where they were excluded, thus creating 8 pairs of corresponding distributions. The mean and maximal change in distribution for each parameter value and bin then allowed to combine everything in one image (Fig. S3). To stay with the example of including low-resolution structures, a mean change of 5.0 for this option in a particular value range meant that the probability of observing such an interface similarity in this range on average changed by 5.0 percent when this option was turned on. Note that this was an exclusively graphical approach to estimate effects, as already changing the number of bins could lead to different values. Since the presentation of results will be kept in this form throughout the rest of this paper, however, it was sufficient in our case.

According to Fig. S3, each parameter can have a considerable effect. Including ΔASA, using 6Å instead of 4Å and high instead of low resolution structures generally stabilized interfaces, i.e. similarities shifted from intermediately high ranges (0.7 to 0.9) to very high conservation (0.9 to 1.0). A different phenomenon arises when using small instead of large clusters: interfaces of small

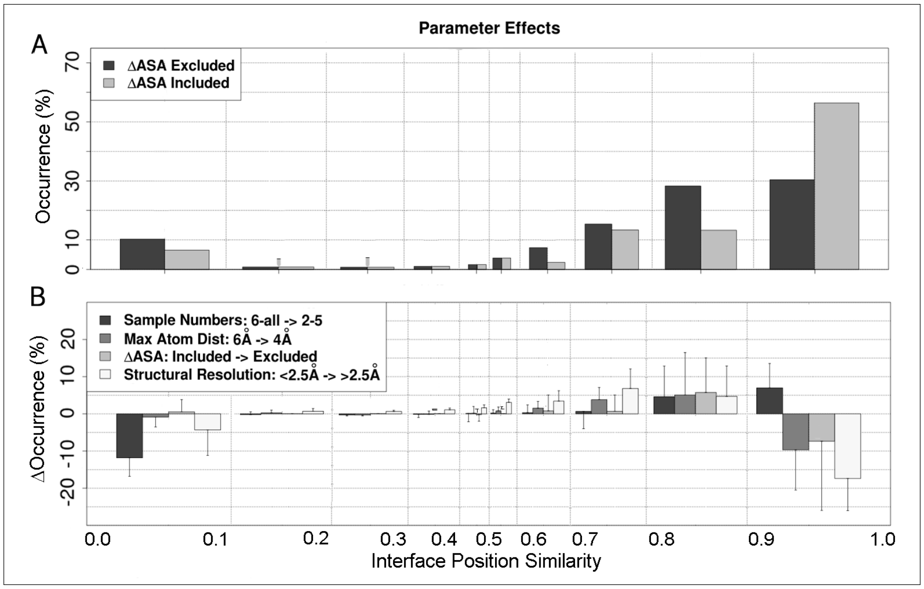

Figure S3: The effect of different parameters on the similarity distribution of interfaces. Both curves were derived with the Interface Position Similarity measure (Section S2.2). **(A)** An example of a distribution change when altering a single parameter value. It corresponds to switching the inclusion of ΔASA on and off (Methods) when considering large complexes with resolutions above 2.5Å and an interaction distance cutoff of 4.0Å. **(B)** The average (thick bars) and maximum (error bars) differences for each parameter and bin with respect to all parameter combinations.

clusters with only 5 or less members tend to be less complementary and more conserved, thus supporting the hypothesis that big complexes encourage alternative binding modes for the same pair of proteins. As they produce more binary structures of the same external interaction, they tend to be found in larger clusters. This is studied in more detail in Section S5.

S3.2 PPI Data Set Properties

Cluster and Complex Sizes

In order to give a better picture of the data at hand (Methods), we compiled histograms of the number of protein chains per complex, external interfaces per Level SameSeq cluster, Level SameSeq clusters per Level SameProt cluster and Level SameProt clusters per Level Interolog cluster (Fig. S4; see Methods and Fig. S1 for a description of the clustering procedure). All plots were calculated with the final data set after filtering (exclusion of bad resolution complexes, small interfaces, ...; see Section S3.1)

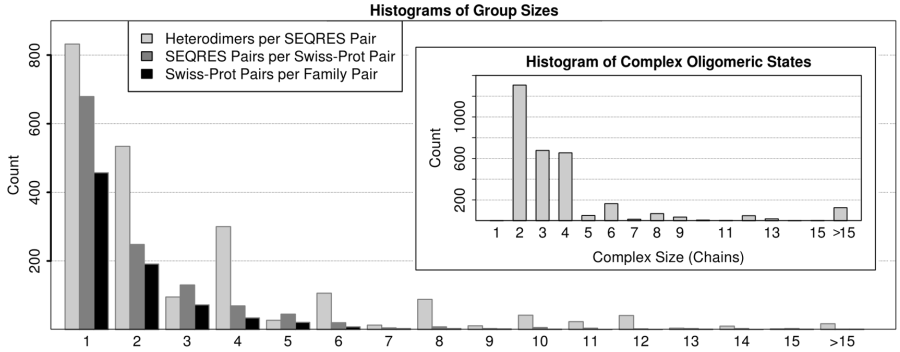

Figure S4: Histograms of various properties of our final data set. We show the distribution of the number of binary interactions per Level SameSeq cluster (heterodimers per SEQRES pair), the number of Level SameSeq clusters per Level SameProt cluster (SEQRES pairs per Swiss-Prot pair) and the number of Level SameProt clusters per Level Interolog cluster (Swiss-Prot pair per Pfam [8] family pair). The inlet additionally displays the histogram of the complex oligomeric states, i.e. the number of protein chains per complex.

The curves in Fig. S4 display typical exponentially decreasing distributions. The distribution of oligomeric states and heterodimers per Level SameSeq cluster are influenced by a natural preference for symmetrical assemblies so that counts for even numbers are overrepresented. Additionally, some larger Level SameSeq cluster sizes are quite frequent (not shown). An overrepresentation of size 17 clusters, e.g., stems from a significant abundance of Cytochrome C Oxidase in the PDB. The effect is already remedied in the second clustering level, however, where exteral interactions are grouped according to the proteins involved.

Interface Sizes

Next, we analyzed the size of interfaces. To this end, we first counted the number of residue-residue interactions in each interface. Then, we calculated the distribution of interface sizes individually for each Level SameSeq cluster. These distributions were subsequently normalized in the same way as a $D_{SameSeq}$ distribution (Methods and Section S1), i.e. we first normalized for overrepresented sequences, then for proteins and then for families. The final distribution is presented in Fig. S5A.

We observe quite a far stretched distribution of interface sizes, as some population exists even beyond 400 residue-residue contacts. Compiling the data with bin size 100, it corresponds to an exponentially decreasing curve, i.e. the smaller the interface, the more frequent. Looking closer at interfaces with 0 to 100 contacts, however, (Fig. S5A, inlet), we see a peak in the range from 40 to 60 residues. The bin from 0 to 20 contacts only plays a minor role.

Structural Similarities

Then, we looked at the Root Mean Square Deviations (RMSDs). As the RMSD typically involves two structures, we implemented it as a standard similarity measure: first, we split the two hetero-dimers under consideration into their four chains. Then, we superimposed corresponding chains, calculated the two RMSDs and returned the average of both (Section S2.8). This allowed us to embed the RMSD into our evaluation framework and observe how values change across different types of comparions ($D_{SameSeq}$ through $D_{Interolog}$; Methods and Section S1). It featured comparisons within clusters (e.g., we calculate one distribution for each Level SameSeq cluster) and across clusters (final distributions $D_{SameSeq}$ to $D_{Interolog}$ are averages over within-cluster distributions). See Fig. S5B for results.

The RMSD distributions impressively show the effects of sequence variations. Comparisons between proteins with the same sequence ($D_{SameSeq}$) most often result in very low RMSD values (0.0Å to 0.5Å). Occurrence of higher values then decreases exponentially and disappears beyond 1.5Å. The distribution of RMSDs between chains from the same protein but different sequences ($D_{SameProt}$) exhibits a clear decrease of high similarity (0.0Å to 0.5Å) and an increase of other ranges up to 2.0Å. When comparing chains from different proteins but the same family, finally, occurrence peaks in the range from 0.5Å to 1.0Å and then steadily declines. As also this distribution stops early for values above 3.0Å, we can say that any $D_{Interolog}$ distribution compares proteins with the same structure, but different sequences.

Function Conservation

We tried to analyze the relationship between protein interactions and protein functions. Unfortunately, large scale function annotations as found, e.g., in Swiss-Prot, in the form of, e.g. Gene Ontology (GO; [2]) terms or Enzyme Commission (EC) numbers, only reach the protein and not the sequence level. Therefore we limited our analysis to the functional diversity found in Level Interolog of our clustering (Methods and Section S1), where proteins are grouped by Pfam [8] families. (Note that Pfam itself aspires to only group functionally related proteins. Hence, we are to some degree comparing different function classification systems.). In this context, we first have to report a negative result: after mapping level Interolog cluster with more than 1 member to experimental GO annotations (evidence codes IDA, IMP, IPI, IGI, IEP, TAS, IC, and EXP), only 26 clusters had more than one functionally annotated protein pair. This was clearly not enough to generally link functional and interfacial diversity. Even a case-by-case reasoning failed: 15 of 26 clusters contained proteins which differed in their annotation already on the first level of the Molecular Function ontology. Curiously, the term leading to by far the most diversity was "binding". This means despite clear evidence of protein binding in the PDB, the experimental evidence had not made it into Swiss-Prot, yet. Manually curating the annotations in the 26 clusters after this finding, we could not find

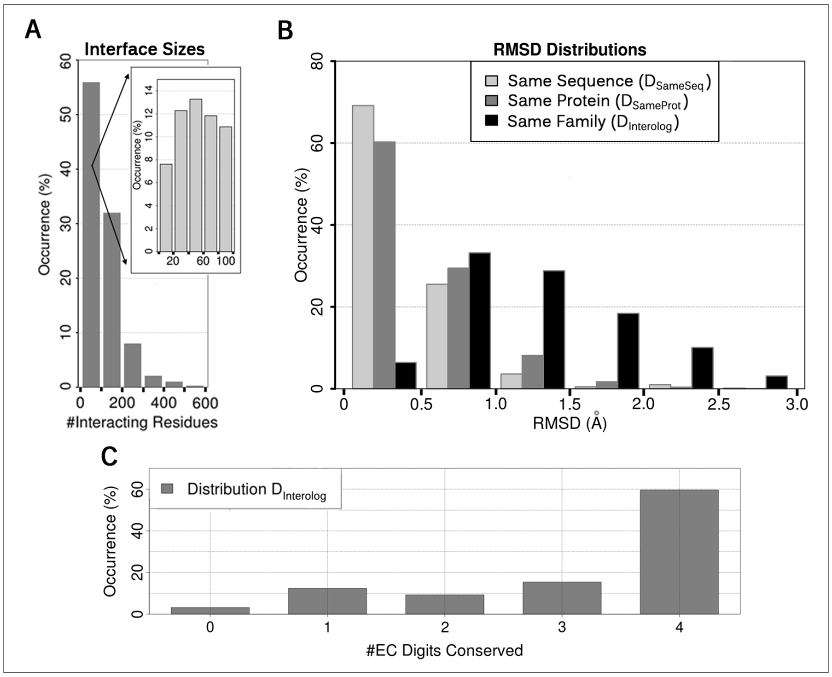

Figure S5: (A) Distribution of interface sizes. We counted the number of residue-residue contacts per interface and calculated the distribution of interface sizes for each Level SameSeq cluster. In this way we could derive distribution $D_{SameSeq}$ like for any other similarity measure (Methods and Section S1). Subfigure (a) shows this distribution first on a rather coarse scale (bin size 100). As it peaks between 40 and 60 contacts, we additionally zoom into the first bin (inlet). **(B) Distributions of RMSDs.** We interpreted the classical Root Mean Square Deviation as a similarity measure and superimposed corresponding chains when comparing two binary heterocomplexes in a Level SameSeq cluster. The two resulting RMSD values were always averaged. This allowed us to calculate $D_{SameSeq}$ through $D_{Interolog}$ (Methods and Section S1). **(C) Function Conservation within Level Interolog clusters.** We analyzed how often and to what degree the EC number between two proteins from the same Pfam family is conserved. The distribution shows the average over all families after averaging the pairwise conservations within each family. This corresponds to the procedure used to calculate the $D_{Interolog}$ distribution in the context of interface similarity measures (Methods and Section S1.2).

clear evidence of functional diversity.

Consequently, we switched from GO annotations to EC numbers, in the hope of more and more complete functional annotations. Indeed, the majority of proteins were annotated with EC numbers, and we could derive a distribution of function conservation in the following way: First, we defined a pairwise function

Actual State	Sensitivity	Incorrect States
1-mer	0/2	2 x 2-mer
2-mer	4/7	2 x 4-mer, 8-mer
4-mer	34/36	2 x 2-mer
6-mer	3/3	-
8-mer	1/1	-
9-mer	29/29	-
12-mer	31/32	6-mer
16-mer	0/2	2 x 8-mer
20-mer	0/1	18-mer
21-mer	4/4	-
24-mer	2/2	-
Overall Accuracy: 90.7±2.7% (108/119)		

Table S1: The accuracy of PISA evaluated with 119 heterocomplexes. "Actual State" refers to the actual oligomeric state of a given complex, "Sensitivity" to the fraction of correctly predicted complexes in the respective state and "Incorrect States" to the oligomeric states the complex was assigned to in case it was predicted incorrectly.

similarity measure: given two proteins, it returns the number of conserved EC number digits. For example, if protein A has EC number 3.4.11.4 and protein B number 3.4.16.1, the measure returns the number 2, because the first two digits are conserved. Calculating the distribution of pairwise functional similarities in this way for each cluster and then averaging over all clusters, we obtained a $D_{Interolog}$ distribution (Methods and Section S1.2). We present it in Fig. S5C. Here, we see that 60% of protein pairs in a cluster have exactly the same EC number. Conservation then sharply drops for 3 and 2 conserved digits, but rises again for 1. This should mainly be due to random effects, however, since complete enzymatic heterogenity (no conserved digits) has the lowest occurrence (3%).

S3.3 On the Accuracy of PISA

Crystallographic methods often do not allow the accurate determination of the biologically relevevant protein assembly, especially in the case of larger complexes. Experimental ways to look at assemblies in the living cells (e.g. cryo electron microscopy) are limited. For our studies, we exclusively used author assigned biological assemblies as annotated in the PDB. In most cases, this means that some interfaces from the aymmetric unit (ASU) have been deemed crystallographic artifacts and that the original complex has been broken down into smaller fragments. Crystallographic interfaces are often determined on the basis of, e.g., interface size (the smaller, the less likely to be relevant) or homol-

ogy (e.g. a protein is similar to another tetramers, thus it is also a tetramer). The PDB provides biologically relevant complexes in the form of downloadable structures, besides the ASUs. They cover about 99% of all high-resolution PDB entries with external interfaces (data not shown) and appear to be quite reliable: in about 100 manual checks whether the author-assigned biological unit as deposited in the PDB was also described as such in the publication introducing the structure, we found only 1 clear mistake.

Despite this high accuracy and coverage, PISA [5], the successor of PQS [3], provides yet another view onto the PDB interactome: it cannot only differentiate between specific and non-specific interfaces, but also re-assemble the fragments of the ASU in order to build the most probable quarternary structure in the living cell. Hence, it might find assemblies which crystallographic methods miss due limitations of experimental methods. We took the opportunity of this work to study its accuracy in context of external interfaces, i.e. interfaces coming from different proteins. While in the majority of cases, there is no experimental data to verify its predictions (which is also why we did not use PISA in the main text), a number of highly accurate biological complexes were available in the PIQSI [7] database.

PIQSI is a manually curated database annotating the oligomeric state of around 15,000 PDB entries (May 2011). Each complex is given an error attribute indicating whether it has the correct oligomeric state. We only considered PIQSI complexes "without errors", thus reducing the number of annotated complexes to 10,000. Unfortunately, only a small fraction of those were heterocomplexes, so that after intersecting PIQSI with our data, only 119 complexes remained for evaluation. Corresponding coordinate files were dowloaded from the PIQSI website. We then compared the oligomeric state of each of those complexes to the most probable complex predicted by PISA. Results are given in Table S1 in a similar way as found in [4].

The evaluation reveals quite a high precision of PISA with an accuracy of 90.7%. This is in line with previous accuracy reports [4]. Note that an erroneous prediction does not automatically lead to distorted similarity distributions for all external interactions of this complex as only a fraction of interfaces should undergo changes in the transition from the incorrect to the correct quaternary structure. Also notice that errors are not systematic over or underestimates of complex sizes so that missing or superfluous interfaces introduced by the wrong oligomeric states should roughly equal each other out with respect to complementarity. To validate these claims, we applied our clustering procedure (Methods and Section S1) to the PIQSI data set and compared the Face Position Similarity distributions to those derived from the PISA data set (distribution $D_{SameSeq}$; Fig. S6A). We did not require two or more PDB entries per external interactions, however, in order to increase the number of interactions for PIQSI. Thus, in absolute terms, curves should be biased towards low similarity.

Due to the small sample size, only a distorted curve could be derived in Fig. S6, but it becomes clear that interfaces are largely the same in both complex sets. Given these estimates, it is unlikely that the more accurate determination of the quaternary state of a protein will have a significant impact on the results.

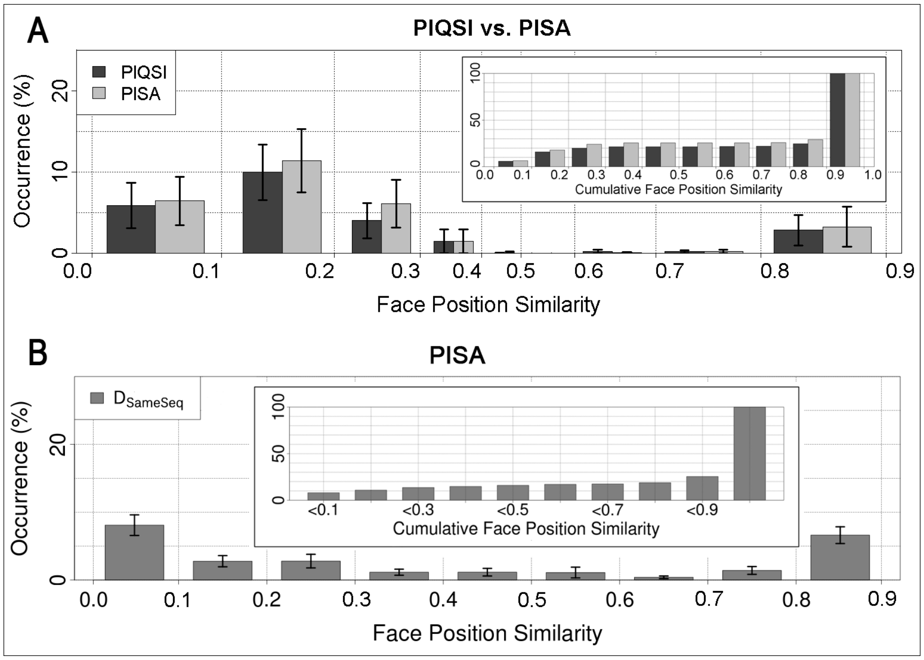

Figure S6: (A) Comparison of Face Position Similarity distributions derived from complexes common to our data and PIQSI. This panel shows the Face Position Similarity Distribution $D_{SameSeq}$ when only comparing complexes common to both PISA and PIQSI. The range 0.9-1.0 is omitted in order to emphasize the other ranges. It is shown again in the inlet. **(B) Comparison of Face Position Similarity distribution $D_{SameSeq}$ on the PISA data set.** Here, we replaced all author assigned complexes in our data set with those predicted by PISA and re-calculated the Face Position Similarity distribution.

In fact, after replacing all author assigned PDB assemblies in our data set with the most probable PISA assemblies and re-filtering and re-clustering all interactions, the according face similarity distribution was mostly within the standard errors (S6B).

S4 Significant Complex Subgroups

There is a possibility that the observed interface variability is confined to particular complex families or subgroups which are known to be overrepresented in the PDB. To address this, we first looked at the occurrence of the 0.9 to 1.0 similarity range in the distribution of each Level Interolog cluster (no sequence divergence [$D_{SameSeq}$]; data not shown). We found a continuum of values, ranging from 0% to 100%. This means there is no particular group of families responsible for

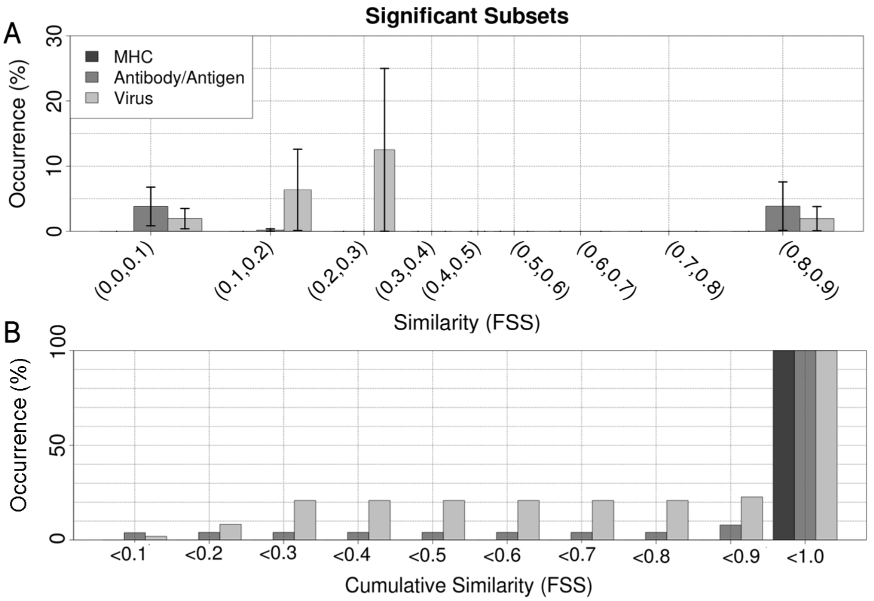

Figure S7: Distributions of interface similarity in particular complex subgroups. In **(A)**, we show the $D_{SameSeq}$ distribution without the range 0.9-1.0. It was omitted due to its overall dominance. Instead, we present it in the corresponding cumulative distribution **(B)**.

the overall observed interface variability. Instead, there are examples for each degree of variability. Furthermore, we found that the size of a family (number of Level SameSeq and Level SameProt clusters in a Level Interolog cluster) does not correlate with its 0.9 to 1.0 bin. Secondly, we queried the PDB for known Virus, Antigen/Antibody and Major Histocompatibility (MHC) complexes and determined the intersection of these subgroups with our data set. This revealed 39 viral, 235 Antigen/Antibody and 198 MHC structures in our data, accounting for 3.0%, 18.2% and 15.4% of all complexes, respectively, and, due to overlap between the sets, together to 21.8%. Distributions for the Face Position Similarity measure ($D_{SameSeq}$; Section S1 and Methods) and each subgroup are given in Fig. S7.

Even though the distributions of the three subgroups substantially differ from the overall distributions, it becomes clear that they are not exclusively responsible for interface variabilities observed in the entire data set. MHC complexes actually appear to be the cause for quite a high fraction of conserved interfaces. In contrast, the Antibody/Antigen subgroup populates the area from 0.0 to 0.9 with 8% of all similarities. In the same range, viral interface similarities are more frequent at a cumulative rate of 21%. Due to small sample sizes, error estimates in all distributions are generally large.

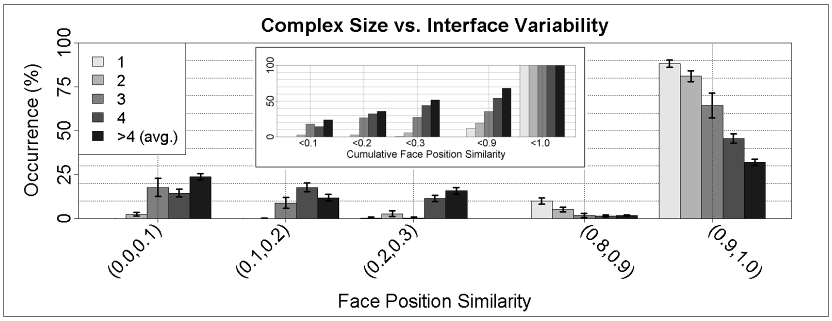

Figure S8: The change of the similarity distribution when restricting comparisons to interfaces with a particular copy number. A given interface corresponds to a certain interaction of two proteins and originates from a particular complex (Fig. 1). We counted how often the interaction occurred in the complex and assigned this number, the interface copy number, to the interface. We repeated this for all interfaces. In the Figure above, we show $D_{SameSeq}$ distributions derived exclusively wth interfaces sharing a particular copy number. This number is depicted by shades of gray: the darker, the higher.

S5 Complex Size vs. Interface Variation

The oligomeric state of a complex should be linked to interface variability, as the necessity for alternative binding increases with the size of the complex. Since higher order complexes can be built from many different proteins, however, it is not so much the plain oligomeric state that should be linked to variability, but rather the number of copies of the same external interaction in one particular assembly. We verified this hypothesis in the following way: first, for a given interface, we determined its corresponding interaction and complex. In Fig. 1, for example, interface X-Y corresponds to interaction S1-S3 and complex C1. Then, we counted how often this interaction occurred in the complex (Fig. 1: S1-S3 occurs twice in complex C1) and assigned this number to the interface (Fig. 1: interface X-Y has copy number 2). This was repeated for all interfaces in our data set. By restricting measurements to interfaces with the same associated copy number, we could then derive one overall similarity distribution $D_{SameSeq}$ for each observed copy number. These distributions were calculated as before with the procedure described in the Methods and Section S1.2. In case of copy number 3, e.g., this meant that we calculated the distribution of a single Level SameSeq cluster (Methods) only with interfaces which come from a complex in which this interaction occurred 3 times. Comparisons between interfaces with different copy numbers were discarded. Due to lack of samples for specific copy numbers above 4, we had to limit this analysis to copy numbers 1 to 4 plus an average over interfaces with a copy number higher than 4. Results are given in Fig. S8.

As can immediately be seen from the high similarity range (0.9 to 1.0),

interface similarity is clearly linked to the copy number of the interaction in the respective complex. Interfaces in dimers are highly conserved, with differences almost exclusively staying in the range from 0.8 to 1.0. High similarity then gradually declines so that the curve for complexes with more than 4 copies of the same interaction shows similarity to an equal distribution.

S6 Distributions of Interface Similarities

S6.1 Face Similarity Distributions

In the following, we present the results for face similarity measures (Fig. S10; measures introduced in Sections S2.4, S2.3 and S2.5). The calculation of the distributions is described in the Methods and Section S1.2. Together with a more detailed discussion, we complement the corresponding paragraph in the Results Section.

Overall, the curves for Convex Hull Overlap are similar to those of L_rms. Note, however, that this measure exclusively compares the interface area and not the entire proteins. Its high sensitivity comes from a comparison of interface shapes and their overlap in volume. Side-chain movement on the edge of an interface, for example, can lead to different interface shapes and thus to lower Convex Hull Overlap.

Sphere Radius Ratio, on the other hand, is the most robust of all measures. This is mainly because it is very difficult to achieve low similarities the way it is calculated (radius of one interface devided by radius of both interfaces combined) and considering the typical proportions of a protein. It is interesting to see that the rough positions of the interfaces on the proteins appear to be conserved even for $D_{Interolog}$.

Interface Composition Similarity compares the amino acid compositions. Also here, dissimilarity is present in all distributions and proofs that differences in terms of residue positions are not due to interface duplications, as expected for example for a protein with two sequentially identical domains. As the measure is also prone to return higher similarities simply by chance (any two interfaces larger than 10 amino acids will have at least one in common), we additionally show the random distribution which was derived by randomly picking interfaces and comparing their compositions. While $D_{SameSeq}$ shows typical values in the area of high interface conservation, lower ranges are not populated. This is in line with the random distributions. In $D_{SameProt}$ and $D_{Interolog}$, the divergence of sequences sets in, because we now allow different amino acids at the same position in the interfaces. The range 0.9-1.0 in $D_{Interolog}$ has the lowest absolute value. Curiously, the influence of sequence divergence and the random distribution merge seamlessly: $D_{Interolog}$ resembles an equal distribution until about a value of 0.5.

The I_rms distribution is quite complex: in the range >4.5Å-4.0Å, we see a difference between distributions similar to L_rms , but with lower overall occurrence. However, the fraction of comparisons with highest I_rms, i.e. where no

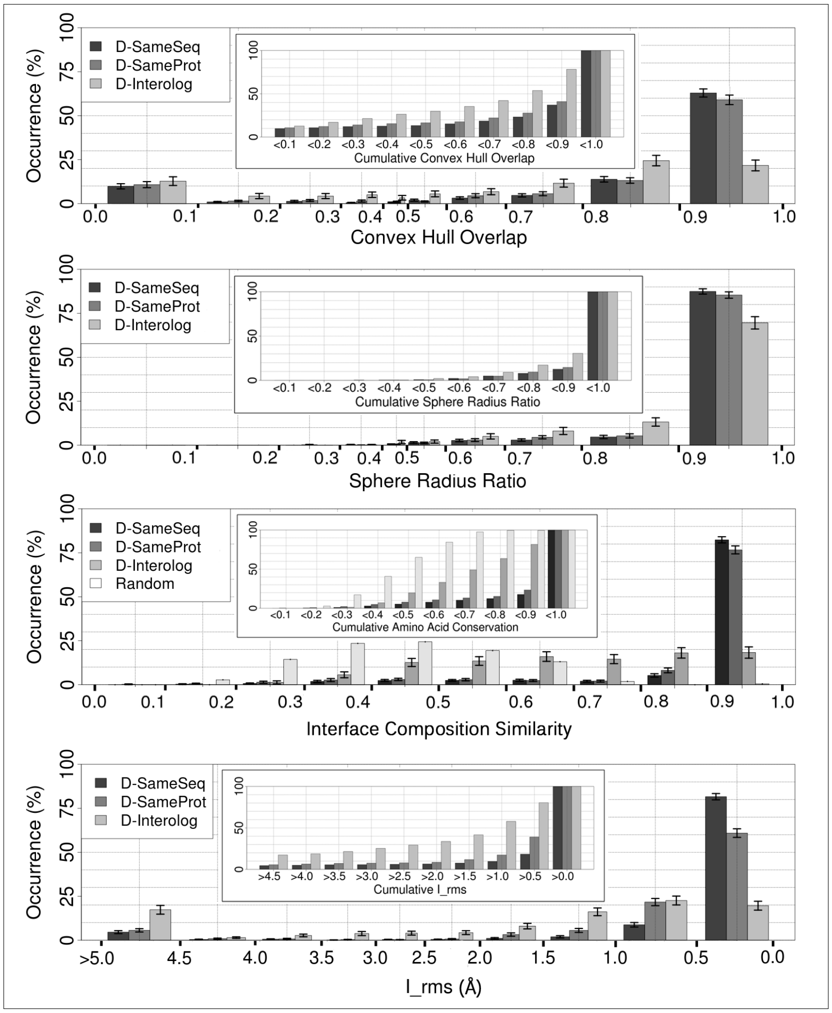

Figure S9: The face similarity distributions for Convex Hull Overlap, Sphere Radius Ratio and Interface Composition Similarity. The random variant of Interface Composition Similarity was derived by repeatedly randomly picking two interfaces from two different clusters in the respective level and applying the measure. For each similarity range and measure, there are three bars. Their order corresponds to the three Distributions $D_{SameSeq}$ to $D_{Interolog}$ (Methods). The inlet in each plot displays the corresponding cumulative distributions.

common interface residues could be found, accounts for only 1-2% *in all three distributions* (not shown). Consequently, their differences must come from comparisons for which common interface residues could be found, but where these residues had very different atom coordinates. This could be due to, e.g., inter-

face rotations, different overlapping binding modes, but also to overall conformational changes (for the I_rms, we included residues in the interface which were as far as 10Å apart). We can hypothesize from this result that 'binding clouds', as shown in the sample structures, should be more frequent in D-Interolog than the other two distributions. Moving on to intermediately low similarities, they seem to be surprisingly rare. One issue is that common interface residues can often be found for only one side of the interaction, i.e. for only one face. In this case, I_rms turns into a comparison of identical fragments of the same protein (family), instead of a comparison of interfaces. Such comparisons usually result in low similarity/high distance ranges (1.0Å- 0.0Å). Similarly, even if all chains have common residues, the exclusion of non-common interfaces residue can lead to low I_rms despite actually different interfaces. Compared to other measures, I_rms should therefore be considered as rather insensitive. Nevertheless, in absolute terms, occurrences of identical interfaces fell well in between the ranges of the other measures.

S6.2 Interface and Domain Similarity Distributions

Now, we show the curves for interface and domain similarity measures (Sections S2.2, S2.6 and S2.7) that were excluded in the main text. As results are overall very similar to those already shown in the context of other measures, we focus on simplicity and only present $D_{SameSeq}$ distributions.

In general, Interface Position Similarity (Fig. S10) draws a similar picture as the face counterpart. As could be expected, though, the chance that a pair of interacting residues is conserved becomes very small when one or both of the interacting faces change according to the Face Position Similarity distributions, thus leading to an increase of high complementarity for Interface Position Similarity compared to Face Position Similarity. Only around 10% of all similarities now lie in the intermediate range between 0.1 and 0.9. Changes in domain numbers or families as measured by Domain Number Ratio and Family Interaction Similarity occur in about 5-15% of all $D_{SameSeq}$ comparisons. We can interpret this observation to imply that interfaces are slightly more similar in terms of this measure than in terms of the others. However, we could also argue that this low difference is partially explained by ambiguities in terms of the domain definitions (and directly connected to this: in terms of the number of domain families), as for instance SCOP and CATH domain assignments differ by about this magnitude [48,49]. In any case, it indicates a steady rate of multi-domain proteins which have learned to interact with their domains in more than one way. Only considering domain-domain interactions, there is a chance we miss parts of the interface between two entire proteins and do not see, for example, that not all domains are always needed for the interaction.

S6.3 Cross-correlations

The full extent to which interfaces vary is best appreciated when cross-correlating multiple measures. The same interface pair might appear identical by one mea-

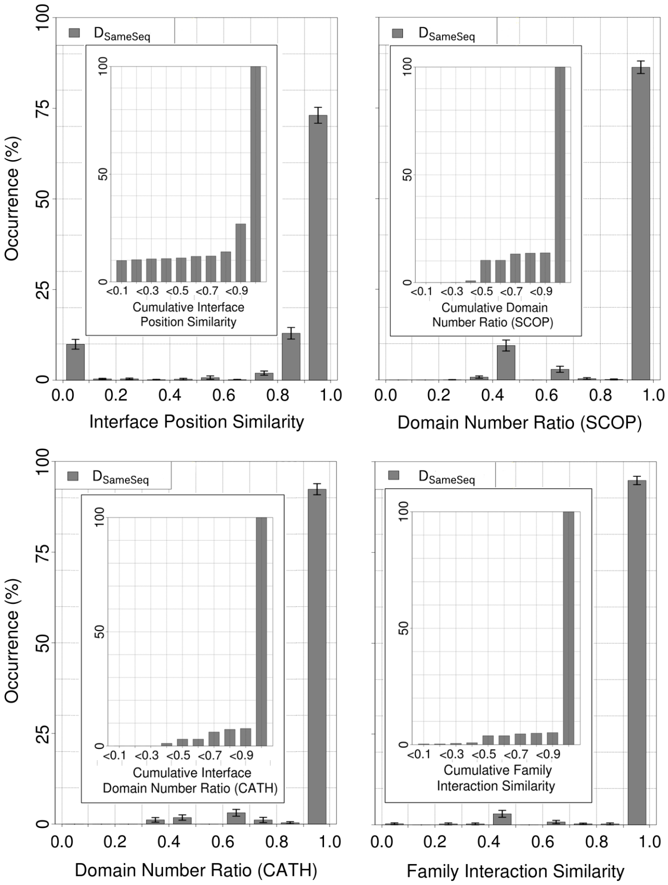

Figure S10: The interface similarity distributions for Interface Position Similarity, Domain Number Ratio (SCOP and CATH) and Family Interaction Similarity. We limited the analysis to distribution $D_{SameSeq}$ for reasons of simplicity. In the context of the Domain Number Ratio, we present the results for both SCOP and CATH. The Family Interaction Similarity was only derived for SCOP. In each plot, the inlet shows the corresponding cumulative distribution.

sure and different by another. To identify such cases, we selected a few pairs of measures and derived the correlation between their distributions. To this end, we no longer calculated the occurrence of a single similarity range (e.g. 0.9 to 1.0) for one particular measure. Instead, we looked at two similarity ranges simultaneously, each corresponding to a different measure. For example, we measured how often the similarity of a pair of interfaces lies in the range between 0.9 and 1.0 according measure 1 while being between 0.1 and 0.2 for measure 2 (Section S1.4 for details). The visualization of the entire distribu-

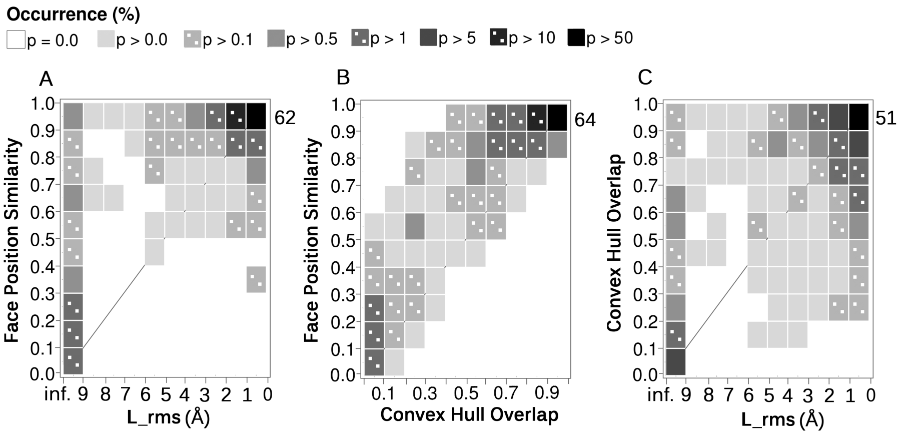

Figure S11: Cross-comparing measures reveals more diversity. Overall, the different measures agree that most repeated measurements yield similar results. However, they often disagree in that one considers a pair of two measurements to be similar while the other measure considers the same to differ. Here, we show three cross-correlations of measures (A-C). Simply put, if the measures agreed, all diagonals would be black, and everything else would be white (blank cells mean that the particular pair of interface similarities has not been observed). The uppermost rightmost cells correspond to very high interface similarity according to both measures and also show the exact percentage of the respective combination. All plots show $D_{SameSeq}$ distributions.

tion, i.e. all possible range combinations, then obviously required three axes: one determining the value of the first measure; one the value of the second measure; and one the probability of observing both similarity values at the same time. Simple matrix plots displayed this: the first measure was assigned to the x-axis and the second measure to the y-axis. The square at a particular x-y coordinate then corresponded to the occurrence of this particular combination of interface similarity: The darker the square, the more often the combination was observed. Hypothetically, these distributions could again be derived for distributions $D_{SameSeq}$ to $D_{Interolog}$. The relations between the measures is already evident for $D_{SameSeq}$, however, so that we limited the analysis to comparisons between interfaces from the same sequence pair ($D_{SameSeq}$). Results are presented in Fig. S11.

If one measure suggests that an interface is identical between repeated observations and another that it differs, which one is right? Clearly, the cross-correlation of measures adds to the view that interfaces have some flexibility to cope with change. In 62% of all comparisons, both Face Position Similarity and L_rms agree that the interfaces between two measurements were largely identical (Fig. S11A). The remaining 38% mainly appeared in ranges above 0.5 in both measure, with a slight tendency of L_rms to be more sensitive than Face

Position Similarity. This trend strikingly manifests itself for L_rms values above 9 (7% of all comparisons): here, we see at least some population in every range of Face Position Similarity. Apparently, conformational changes of the entire protein can lead to high RMSDs, but preserve the interfaces to a point where no interface residue changes are detectable. The matrix comparison between Convex Hull Overlap and L_rms shows a similar trend, but is more pronounced (Fig. S11C). Now, we observe almost any pair of similarities. Again, highest L_rms is often accompagnied by intermediate Convex Hull Overlap. Note that both measures agree on conservation of an interface pair in only 51% of all cases. This means that there is a 49% chance that the same interfaces from two different PDBs will differ from each other by at least one measure. Comparing Face Position Similarity and Convex Hull Overlap, finally, we see that Convex Hull Overlap is significantly more sensitive (Fig. S11B). It assigns lower values to the same interface pair in about 27% of all cases, while Face Position Similarity does the same in only 2%. In case the two measures differ, the difference is usually not large: most values are close to the diagonal and there are no cases where one measure assigns highest difference and the other highest similarity.

S7 Additional Sample Structures

In the following, we discuss cases of differing interfaces for the same pair of sequences. They add to the picture of binding diversity.

Yersinia pestis (Fig. S12A) represents one of the very few cases of clear alternative binding between two interfaces with copy number 1 (Section S5). All measures agree in this finding. The antigen usually forms fibers by repeatedly binding to itself. In the two structures displayed here, this has been disabled by mutating the N terminus in various ways. What remains are two original interactions with a chaperone protein, representing snapshots of different stages in the fiber assembly process. Note that 1P5U has actually three chains with two corresponding to the antigen, but only one of them also corresponds to the N-modified variant present in the binary structure 1P5V.

Choleraholotoxin is an extreme case of an interaction with Face Position Similarities almost exclusively lying in the intermediate range 0.1-0.9 (Fig. S12E, Fig. S13C,D). The shorter B chain forms a cyclic homo-pentamer with a pore in the middle. The A subunit occupies this space with a terminal loop. This results in several different binding positions of a single B chain on the A subunit in one PDB structure. Furthermore, another structure of the same complex reveals that the pentamer exhibits some translational freedom with respect to the A subunit, leading to even more interface diversity. These types of rotational interfaces can generally be found by cross-correlating the measures Sphere Radius Ratio and Face Position Similarity. A high Sphere Radius Ratio suggests that interface sizes and locations are conserved. If the Face Position Similarity is low, change must therefore come from a rotation around a central axis. Only in this way, we preserve the radius and position and yet change the residues of the face.

For hemoglobin, (Fig. S12C), a clustering should easily interpret the two big interface 'cloud' as such and identify two distinct binding modes. This is further supported by the distribution of pairwise interface similarities (Fig. S13C) that is mainly populated either in regions of low (0.0-0.2) or high similarity (0.9-1.0). Closer inspection, however, reveals that the clouds are by no means biologically irrelevant: A main contributor to their variety for example is the change of hemoglobin from the T to the R conformation when releasing or binding oxygen. A typical clustering would not only miss this conformational change, but also its seemingly continuous nature with many intermediate states. Note, however, that a detailed functional annotation of each interface according to the conformational state could improve that.

We discuss Cytochrome BC1 and Type IV collagen in the caption of Fig. S12 and RuBisCO in the next Section.

Difference in Number of Interacting Families

Over 7% of all interface comparisons suggest a difference in the domain families that interact (Fig. S10). Two examples illustrate the above conclusion that many of those are limits in the reliability of our domain/family definition. The first is RuBisCO (Fig. S13A) that has a short chain (single domain, single domain family). It has evolved three clearly distinct binding positions on the large chain (2 domains, 2 domain families). Two of these faces fall on the same domain; the third falls on a different domain. Consequently, we see two different family pairings in the same pair of proteins. The second example is the complex of aldolase-dehydrogenase (Fig. S13B). The two aldolase domains both contribute almost equally to one interface while on the other interface, only the catalytic domain interacts with the dehydrogenase. The same pattern is true for the comparisons between the faces on the two-domain dehydrogenase (not shown).

S8 Functions of Families With and Without Interface Variability

We collected interacting families which show absolutely no sign of interface variability (0.9-1.0 bin in distribution $D_{Interolog}$ at 100% with Face Position Similarity). Then, we determinded the GO terms of the proteins in each of these family pairs and counted how many family pairs were associated with a particular GO term. We show the results in the first half of the following table. Then, we performed the same analysis for family pairs which show very high interface variability (0.0-0.5 bins in distribution D-Interolog sum up to 100% with Face Position Similarity). In both tables, the column "Unique" indicates that the respective GO term was only found in that particular group of family pairs. Terms which only appeared once in either group are not shown.

References

[1] C. Barber, K. Henrick, D. Dobkin, and H. Huhdanpaa. The Quickhull Algorithm for Convex Hulls. *ACM Transactions on Mathematical Software*, 22(4):469–483, 1996.

[2] T. G. O. Consortium. Gene ontology: Tool for the unification of biology. *Nature Genetics*, 25(1):25–29, 2000.

[3] K. Henrick and J. M. Thornton. Pqs: a protein quaternary structure file server. *Trends in Biochemical Sciences*, 23(9):358 – 361, 1998.

[4] E. Krissinel and K. Henrick. Detection of Protein Assemblies in Crystals. *Lecture Notes in Computer Science*, 3695:163–174, 2005.

[5] E. Krissinel and K. Henrick. Inference of macromolecular assemblies from crystalline state. *Journal of Molecular Biology*, 372(3):774 – 797, 2007.

[6] M. F. Lensink, R. Mndez, and S. J. Wodak. *Proteins: Structure, Function, and Bioinformatics*, 69(4):704–718, 2007.

[7] E. D. Levy. PiQSi: Protein Quaternary Structure Investigation. *Structure*, 15(11):1364–1367, 2007.

[8] M. Punta, P. C. Coggill, R. Y. Eberhardt, J. Mistry, J. Tate, C. Boursnell, N. Pang, K. Forslund, G. Ceric, J. Clements, A. Heger, L. Holm, E. L. L. Sonnhammer, S. R. Eddy, A. Bateman, and R. D. Finn. The pfam protein families database. *Nucleic Acids Research*, 40(D1):D290–D301, 2012.

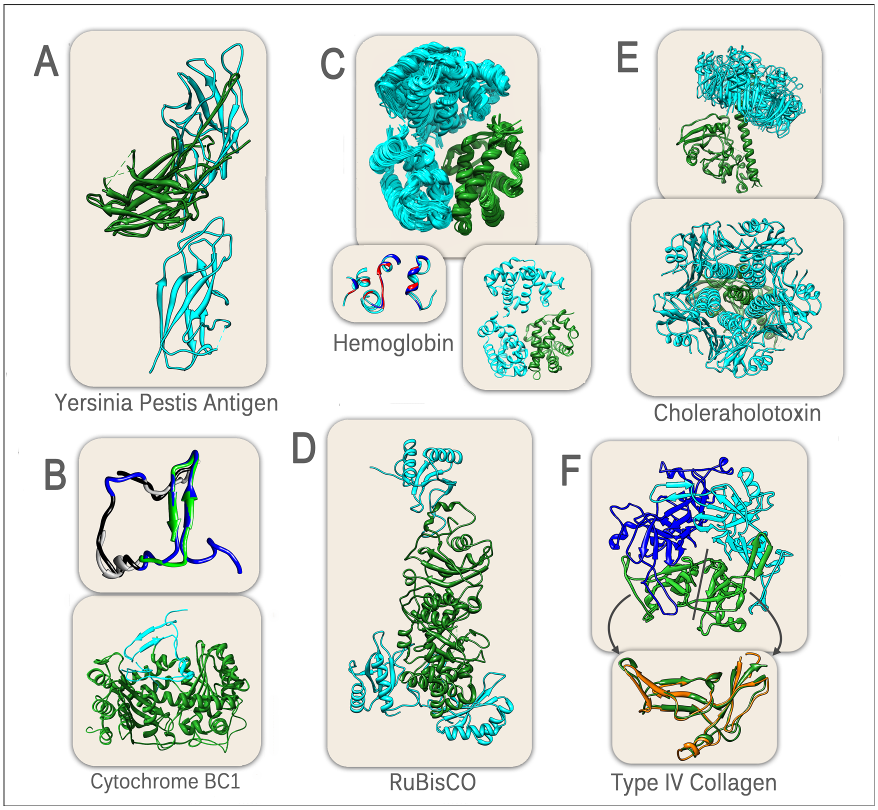

Figure S12: Six additional interactions with surprising interface variety. Each panel (A-F) shows superimpositions of multiple heterodimers with sequences X (dark green, note: all green have RMSD<1Å to each other) and Y. The interacting chains Y were subject to the same geometric translations as their X counterpart and are displayed in cyan. **(A)** The rare case (Section S5) of large interface differences when looking at two structures with interface copy number 1 (1P5U, 1P5V). **(B)** Example for 'low Interface Position Similarity with high Face Position Similarity' (1PP9, 2FYU): lower frame: comparison of two structures of Iron-sulfur subunit precursor (cyan) interacting with core protein 1 (green); upper frame: iron-sulfur subunit precursor superimposition in detail; black and gray: identical subsequences at different spatial locations; green and blue: different subsequences at same spatial location. **(C)** Superimposition of 251 Hemoglobin complexes (e.g. 1A3N); lower left side frame: superposition of two sample faces of upper interface cloud; face residues (blue and red) colored by chain; lower right side frame: one sample complex of each interface cloud; also see Fig. S13C **(D)** Ribulose-1,5-bisphosphate carboxylase oxygenase (1AA1): three highly distinct binding positions; also see Fig. S13A **(E)** Even distribution of Face Position Similarity vs. constantly high Sphere Radius Ratio (1S5C, 1S5D); upper frame: side view; lower frame: view from top; also see Fig. S13 **(F)** High Amino Acid Coupling vs. low Interface Position Similarity (1M3D, 1T61); upper frame: X consists of two homologous domains (domain boundary indicated by separating line) and interacts with Y at two different positions; dark blue used instead of cyan to show chain boundaries; lower frame: domains of X superimposed (domains highly homologous; sequences not shown).

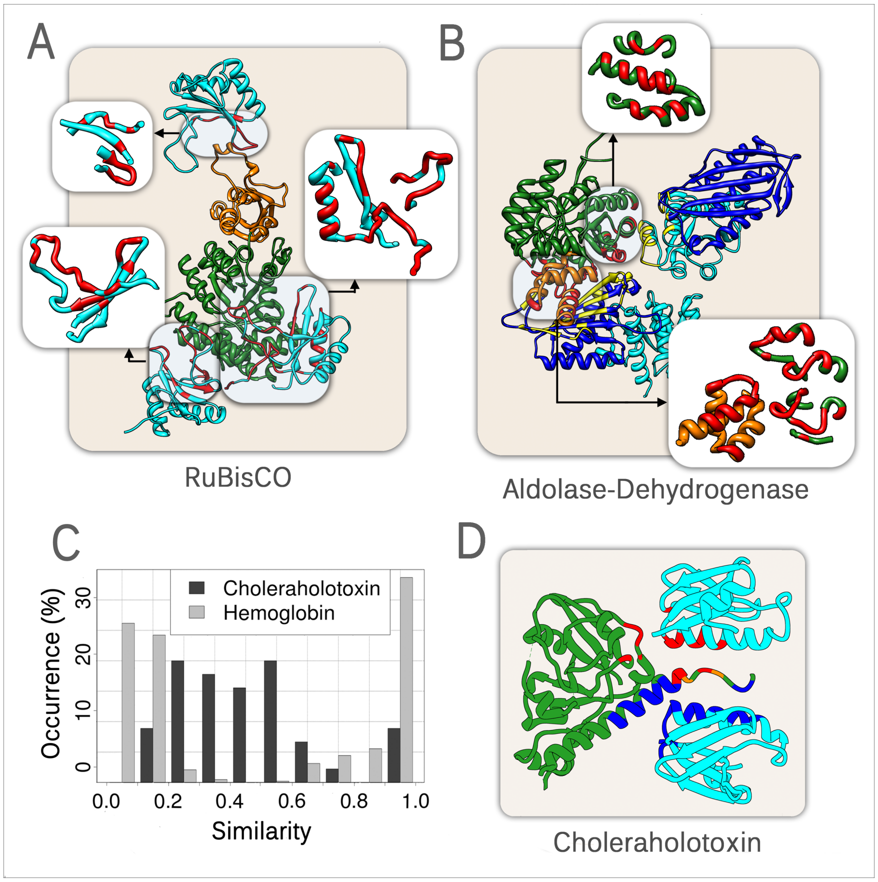

Figure S13: Multi-domain proteins interacting through different SCOP families and difficult cases of interface clustering. Colors as in Fig. S12; exceptions: second domain of X is orange, second domain of Y is blue; interface residues in red. **(A)** RuBisCO from Fig. S12B; interfaces magnified; the short chain (cyan; one domain) has two binding positions on the green domain of the large chain and one on the orange domain. **(B)** Aldolase-Dehydrogenase complex (1NVM); Aldolase domains: green and orange; Dehydrogenase domains: cyan and blue; Aldolase faces magnified; in the first interaction, only the green domain contributes to the face (upper frame); in the second, both the green and the orange domain are involved (lower frame). **(C)** Comparison of the Face Position Similarity distribution of two Level SameSeq groups corresponding to Hemoglobin (Fig. S12C) and Choleraholotoxin (Fig. S12E, S13D). **(D)** Superimposition of two diverse Choleraholotoxin interactions; created from Fig. S12E by removing all but two Y chains.

GO Number	GO Term	Family Pairs (max. 17)	Unique
GO:0003674	molecular function	16	No
GO:0005488	binding	16	No
GO:0043169	cation binding	11	No
GO:0043167	ion binding	11	No
GO:0046906	tetrapyrrole binding	7	Yes
GO:0051540	metal cluster binding	6	No
GO:0016787	hydrolase activity	6	No
GO:0003824	catalytic activity	6	No
GO:0051536	iron-sulfur cluster binding	6	No
GO:0004175	endopeptidase activity	5	No
GO:0008233	peptidase activity	5	No
GO:0046872	metal ion binding	5	No
GO:0070011	peptidase activity, acting on L-amino acid peptides	5	No
GO:0046914	transition metal ion binding	4	No
GO:0030234	enzyme regulator activity	3	No
GO:0030246	carbohydrate binding	2	Yes
GO:0008236	serine-type peptidase activity	2	Yes
GO:0004857	enzyme inhibitor activity	2	No
GO:0017171	serine hydrolase activity	2	Yes
GO:0015077	monovalent inorg. cation transmembr. transp. act.	2	No
GO:0070003	threonine-type peptidase activity	2	Yes
GO:0005506	iron ion binding	2	Yes
GO:0005215	transporter activity	2	No
GO:0008324	cation transmembrane transporter activity	2	No
GO:0022891	substrate-specific transmembrane transporter activity	2	No
GO:0022890	inorganic cation transmembrane transporter activity	2	No
GO:0022892	substrate-specific transporter activity	2	No
GO:0015078	hydrogen ion transmembrane transporter activity	2	No
GO:0015075	ion transmembrane transporter activity	2	No
GO:0022857	transmembrane transporter activity	2	No
GO:0005515	protein binding	2	No

Table S2: GO Terms of families with exclusively alternative interfaces

GO Number	GO Term	Family Pairs (max. 18)	Unique
GO:0003674	molecular function	17	No
GO:0005488	binding	16	No
GO:0043169	cation binding	11	No
GO:0043167	ion binding	11	No
GO:0003824	catalytic activity	10	No
GO:0005515	protein binding	10	No
GO:0046872	metal ion binding	9	No
GO:0016787	hydrolase activity	6	No
GO:0000166	nucleotide binding	5	No
GO:0017076	purine nucleotide binding	5	No
GO:0008092	cytoskeletal protein binding	5	Yes
GO:0030554	adenyl nucleotide binding	5	No
GO:0001883	purine nucleoside binding	5	No
GO:0001882	nucleoside binding	5	No
GO:0046914	transition metal ion binding	4	No
GO:0032559	adenyl ribonucleotide binding	4	No
GO:0032555	purine ribonucleotide binding	4	No
GO:0032553	ribonucleotide binding	4	No
GO:0016491	oxidoreductase activity	4	No
GO:0008233	peptidase activity	3	No
GO:0019899	enzyme binding	3	No
GO:0005102	receptor binding	3	Yes
GO:0030528	transcription regulator activity	3	Yes
GO:0030234	enzyme regulator activity	3	No
GO:0070011	peptidase activity, acting on L-amino acid peptides	3	No
GO:0004866	endopeptidase inhibitor activity	2	No
GO:0060089	molecular transducer activity	2	No
GO:0003712	transcription cofactor activity	2	Yes
GO:0019900	kinase binding	2	Yes
GO:0019902	phosphatase binding	2	Yes
GO:0044212	transcription regulatory region DNA binding	2	Yes
GO:0016563	transcription activator activity	2	Yes
GO:0003676	nucleic acid binding	2	No
GO:0003677	DNA binding	2	Yes
GO:0043565	sequence-specific DNA binding	2	Yes
GO:0022891	substrate-specific transmembr. transp. act.	2	No
GO:0022892	substrate-specific transporter activity	2	No
GO:0051536	iron-sulfur cluster binding	2	No
GO:0015075	ion transmembrane transporter activity	2	No
GO:0046983	protein dimerization activity	2	No
GO:0004857	enzyme inhibitor activity	2	No
GO:0008237	metallopeptidase activity	2	No
GO:0042802	identical protein binding	2	No
GO:0005215	transporter activity	2	No
GO:0008134	transcription factor binding	2	Yes
GO:0008324	cation transmembrane transporter activity	2	No
GO:0048037	cofactor binding	2	Yes
GO:0030414	peptidase inhibitor activity	2	No
GO:0004175	endopeptidase activity	2	No
GO:0051540	metal cluster binding	2	No
GO:0003702	RNA polymerase II transcription factor activity	2	Yes
GO:0022857	transmembrane transporter activity	2	No

Table S3: GO Terms of families without interface variability
